# Identification and characterization of the siderochelin biosynthetic gene cluster *via* coculture

**DOI:** 10.1128/mbio.01871-24

**Published:** 2024-08-27

**Authors:** Adam J. Schaenzer, Wenliang Wang, Dirk Hackenberger, Gerard D. Wright

**Affiliations:** 1 Michael G. DeGroote Institute for Infectious Disease Research, McMaster University, Hamilton, Ontario, Canada; 2 Department of Biochemistry and Biomedical Sciences, McMaster University, Hamilton, Ontario, Canada; MedImmune, Gaithersburg, Maryland, USA

**Keywords:** coculture, siderophores, actinomycetes, iron

## Abstract

Many microbial biosynthetic gene clusters (BGCs) are inactive under standard laboratory conditions, making characterization of their products difficult. Silent BGCs are likely activated by specific cues in their natural environment, such as the presence of competitors. Growth conditions such as coculture with other microbes, which more closely mimic natural environments, are practical strategies for inducing silent BGCs. Here, we utilize coculture to activate BGCs in nine actinobacteria strains. We observed increased production of the ferrous siderophores siderochelin A and B during coculture of *Amycolatopsis* strain WAC04611 and *Tsukamurella* strain WAC06889b. Furthermore, we identified the siderochelin BGC in WAC04611 and discovered that the GntR-family transcription factor *sidR3* represses siderochelin production. Deletion of the predicted aminotransferase *sidA* abolished production of the carboxamides siderochelin A/B and led to the accumulation of the carboxylate siderochelin D. Finally, we deleted the predicted hydroxylase *sidB* and established that it is essential for siderochelin production. Our findings show that microbial coculture can successfully activate silent BGCs and lead to the discovery and characterization of unknown BGCs for molecules like siderochelin.

**IMPORTANCE:**

Siderophores are vital iron-acquisition elements required by microbes for survival in a variety of environments. Furthermore, many siderophores are essential for the virulence of various human pathogens, making them a possible target for antibacterials. The significance of our work is in the identification and characterization of the previously unknown BGC for the siderophore siderochelin. Our work adds to the growing knowledge of siderophore biosynthesis, which may aid in the future development of siderophore-targeting pharmaceuticals and inform on the ecological roles of these compounds. Furthermore, our work demonstrates that combining microbial coculture with metabolomics is a valuable strategy for identifying upregulated compounds and their BGCs.

## INTRODUCTION

Secondary or specialized metabolites (i.e., natural products) are invaluable assets for the pharmaceutical, agricultural, cosmetic, and food industries. Although difficult to precisely enumerate, the number of known secondary metabolites is estimated to be in the hundreds of thousands and is increasing yearly (1). Bacteria and fungi are exceptionally gifted producers of secondary metabolites, with the prolific *Streptomyces* genus alone frequently possessing 20–70 secondary metabolite-encoding biosynthetic gene clusters (BGCs) per genome (2). However, many of these BGCs remain silent under standard laboratory conditions, hindering the characterization of their corresponding products.

Traditional microbial natural product discovery platforms involve creating extracts from producer strains grown in monoculture under idealized conditions, e.g., rich carbon and nitrogen sources. However, microbes rarely encounter such privileged circumstances in nature. To survive in the wild, microorganisms must rapidly respond to extreme fluctuations in environmental conditions and compete with rivals over limited resources. Under these conditions, secondary metabolites serve various survival functions, including intra-/inter-species communication, metal acquisition, and biological weapons (3). It stands to reason that producing these costly metabolites would be tightly restricted, expressed only under stressful conditions for “just in time” delivery. As such, growth conditions that mimic these environments, such as stressing with chemical elicitors [reviewed in (4)] or the coculture of multiple organisms [reviewed in (5, 6)], have gained traction as practical strategies to activate silent BGCs. In coculture, competition over iron acquisition is an example of a driving factor in the activation of secondary metabolite production (7).

Iron is an essential micronutrient for almost all life. However, it predominantly exists in nature in the insoluble ferric (Fe^3+^) form, which is not readily bioavailable. Microorganisms have evolved small molecules called siderophores and their cognate receptors to solubilize and retrieve environmental iron for their own use. Competition over limited iron has led to extensive diversification of siderophores; over 500 have been structurally characterized (8). Interest in siderophore biosynthesis has increased due to their requirement for the virulence of many pathogens, making the production of siderophores a potential antivirulence target (9). Although the biosynthesis of some siderophores has been well characterized, many others remain unstudied.

Here, we present the results of a microbial coculture screen to identify overproduced secondary metabolites in actinomycetes that reveal siderophores to be highly upregulated when bacteria are cocultured. Furthermore, we identify and perform the characterization of the BGC for the ferrous iron siderophore siderochelin, a compound found to be overproduced in our coculture screen.

## RESULTS

### Siderophores are highly upregulated in microbial coculture

**Fig 1:**
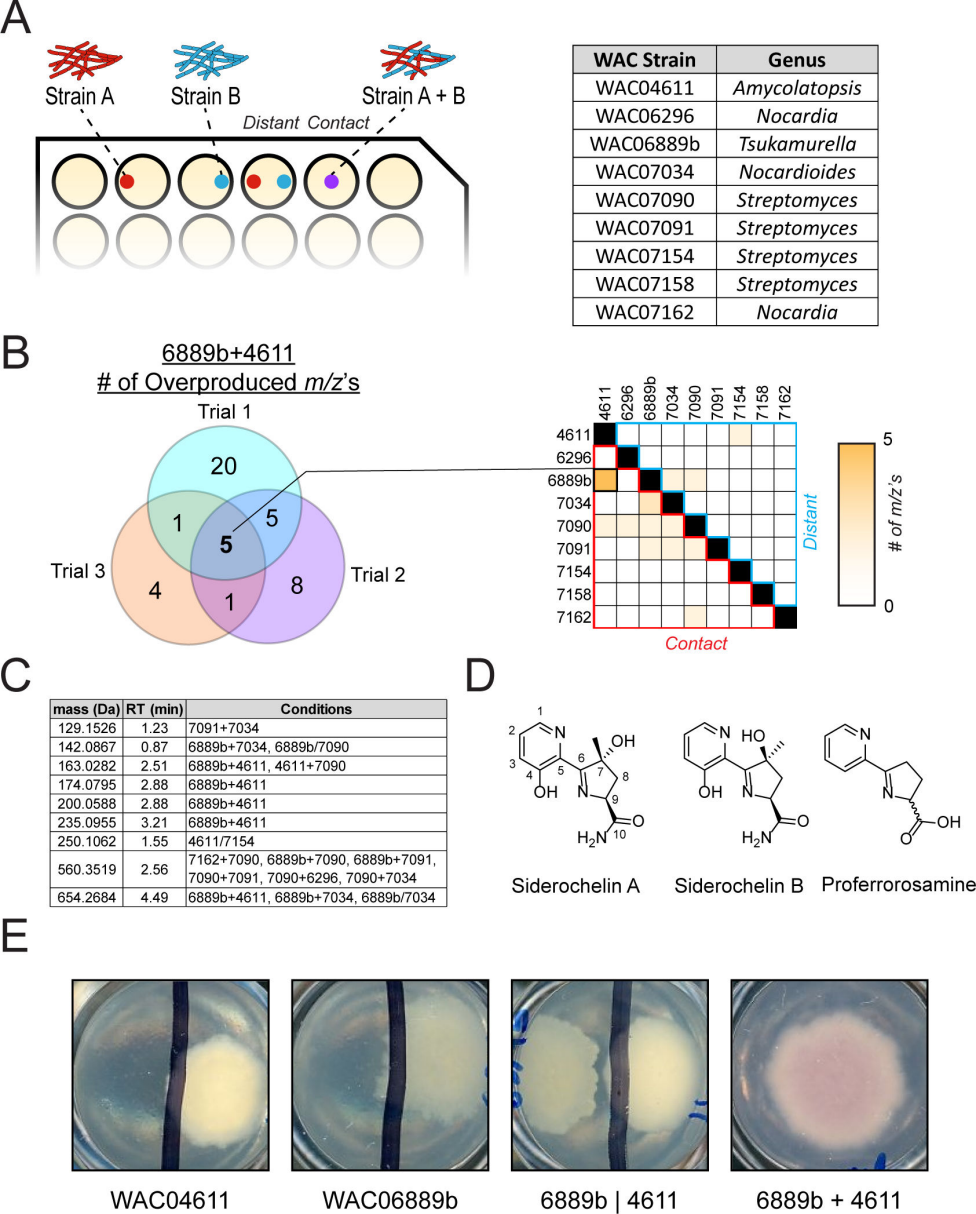
Coculture screen identifies siderochelin as an overproduced metabolite. (**A**) (Left) Schematic of coculture plate. (Right) List of bacterial strains used in the coculture experiments. (**B**) (Left) Number of masses that were overproduced in the 6889b + 4611 coculture relative to either constituent strain alone. Five masses were consistently overexpressed in all three trials. (Right) Number of m/z’s that were overproduced consistently across three biological replicates for each coculture condition. “Distant” denotes pairs that were grown in the same well but at opposite ends of the well. “Contact” denotes pairs that were mixed together and grown in contact with each other. Data are the average of three biological trials. (**C**) Table of upregulated masses from panel “B.” Pairs separated by a “/” denote the “Distant” growth arrangement while pairs separated by a “+” denote the “Contact” growth pattern. (**D**) Structures of siderochelin A, siderochelin B, and proferrorosamine. (**E**) Coloration of agar with different inoculuma.

To explore interactions between competing bacteria, nine actinomycete strains isolated from a single Nigerian soil sample were grown on Bennett’s agar in pairs either as mixed cultures (“contact”) or at opposite ends of a well in a 24-well plate (“distant”) (Fig. 1A). Strains were selected from the same soil sample to increase the likelihood of ecologically relevant interactions. After 9 days of growth, methanolic extracts were prepared from each well and analyzed by metabolomics. Compounds with *m/z*’s that were enriched ≥10-fold relative to either constituent strain alone were considered hits. Although the results were heterogeneous between biological replicates, cultures with constituents in direct contact reproducibly produced more highly enriched *m/z*’s compared with spatially close cultures without physical contact (Fig. S1). Nine masses were consistently overproduced across the three biological replicates, four of which were identified in multiple coculture conditions (Fig. 1B and C). The most prevalent mass, 560.3519 Da, was identified in six different coculture conditions and determined to be the siderophore desferrioxamine B via comparison to a laboratory standard. In agreement with this finding, each coculture condition with this overproduced mass contained at least one constituent strain that possesses an intact desferrioxamine BGC (WAC07034, WAC07090, WAC07091, and WAC07158).

Of all the coculture conditions tested, the *Amycolatopsis sp*. WAC04611, when grown in contact with the *Tsukamurella sp*. WAC06889b, produced the most consistent results with five overproduced masses, three of which were unique to this coculture condition. One of the unique masses, 235.0995 Da, matched that of siderochelin A or B (expected mass 235.0957 Da, mass error 16.2 ppm) from the Atlas of Natural Products (Fig. 1D) (10). The siderochelins are a family of siderophores produced by *Nocardia* and *Amycolatopsis* species that deviate from typical siderophores by preferentially chelating Fe^2+^ over Fe^3+^, a characteristic shared by the collismycins, caerulomycins, and proferrorosamine (11–15). Furthermore, the WAC06889b + WAC04611 “contact” coculture condition uniquely turned pink during our experiments, a color characteristic of the presence of a siderochelin or a similar siderophore (Fig. 1E). Mass-guided purification and nuclear magnetic resonance spectroscopy (NMR) analysis of purified compound confirmed the presence of a siderochelin, based on comparisons of our ^1^H NMR to the spectra displayed in Liu *et al*. (11) as well as our own extensive 2D NMR analysis (Fig. S2). Although our NMR spectra resembled those in the literature, there remained ambiguity around the chiral center at C7 of the dehydropyrrole. Tandem mass spectrometry(MS/MS) experiments on the purified sample revealed a major and minor peak with identical MS/MS fragmentation patterns (Fig. S3). Our NMR and MS/MS data suggest that the purified sample is a racemic mixture of siderochelins A and B. Addition of Fe_2_SO_4_ to our racemic siderochelin sample resulted in deep purplish-pink color and the appearance of an *m/z* of 525.1183 (retention time 0.81 minutes), consistent with the formation of a complex consisting of two siderochelin molecules and a single iron atom.

### Identification of the siderochelin biosynthetic gene cluster

**Fig 2:**
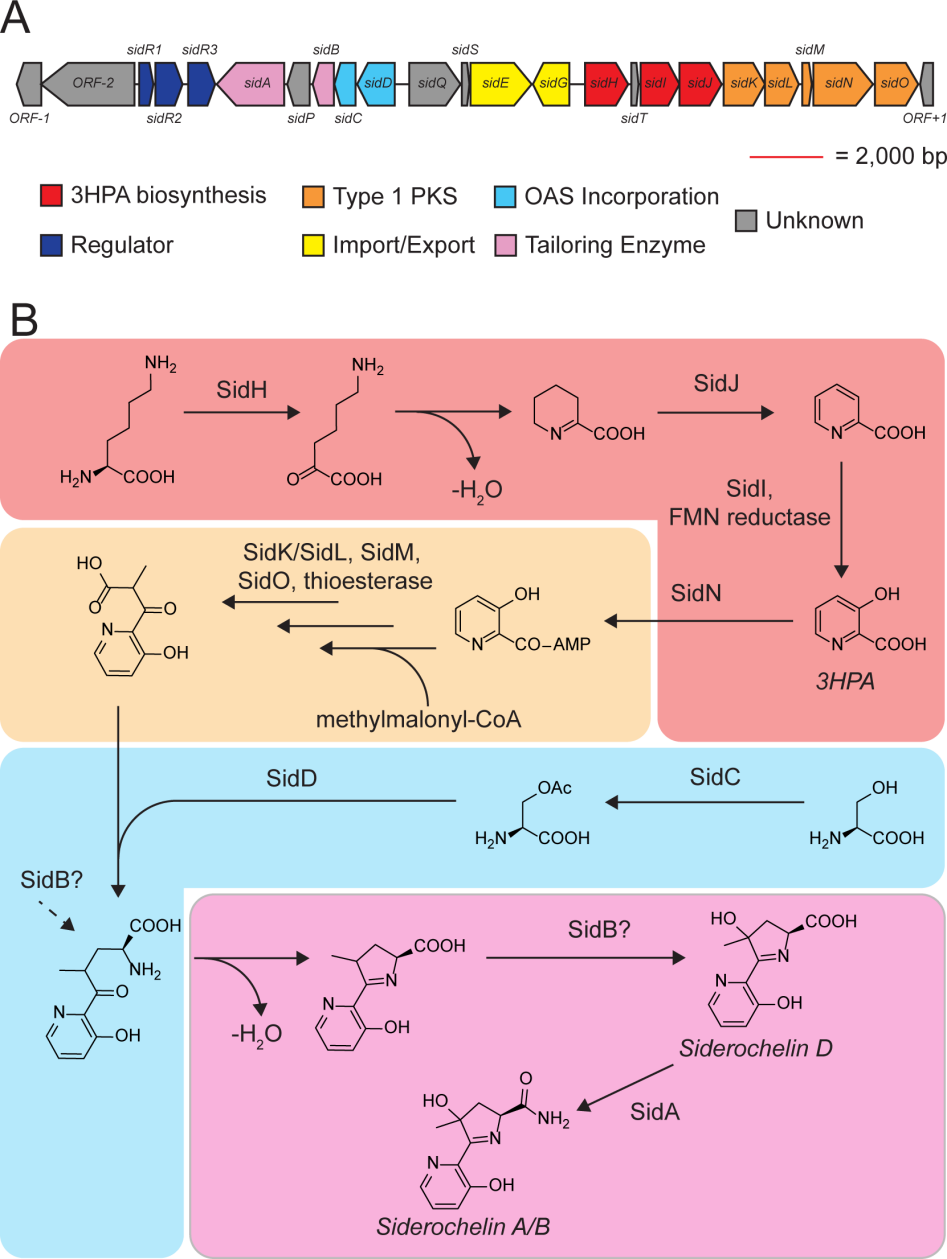
Identification of the siderochelin BGC. (**A**) Schematic of the BGC captured from WAC04611. Open reading frames (ORFs) are colored by predicted functions. 3HPA: 3-hydroxypicolinic acid; OAS: *O*-acetylserine. (**B**) Proposed siderochelin biosynthesis pathway based on Born et al (route A) (16). Red box denotes 3HPA synthesis, orange box denotes PKS-catalyzed incorporation of methylmalonyl-CoA, blue box denotes OAS production and incorporation into scaffold, and pink box denotes dihydropyrrole ring closure and tailoring reactions. Chirality around C7 is displayed ambiguously due to the mechanism and timing of racemization between siderochelin A and B being unknown. Hydroxylation of the dihydropyrrole may occur before or after dihydropyrrole ring closure (denoted by “SidB?”).

Although the siderochelins were first discovered in 1981 (11), their biosynthesis is unexplored. As an entry point to identifying the siderochelin BGC, we compared the structures of the siderochelins with that of proferrorosamine, a molecule with a similar structure and a known BGC (Fig. 1D). In *Erwinia rhapontici*, the proposed biosynthesis of proferrorosamine is initiated by the production of picolinic acid, which is extended by a single malonyl-CoA *via* a type I polyketide synthase (type I PKS). *O*-Acetylserine is next appended to the molecule by the pyridoxal phosphate (PLP)-dependent enzyme RosC, followed by spontaneous ring closure to generate a dihydropyrrole (16). If siderochelin is produced in a similar manner, we expect the siderochelin BGC to possess genes required to produce 3-hydroxypicolinic acid (3HPA) rather than picolinic acid. Using antiSMASH (17), we identified a BGC in WAC04611, which contained the expected genes for 3HPA biosynthesis (*sidHIJ*; Fig. 2A; Fig. S4). Further analysis of the BGC revealed a type I PKS with a predicted single methylmalonyl-CoA module (*sidKLMNO*), genes for *O*-acetylserine production (*sidC*), and a PLP-dependent enzyme homologous to *rosC* (*sidD*), expected to generate dehydroalanine from *O*-acetylserine, which reacts with the decarboxylated PKS product. Intramolecular imine formation completes the core molecular scaffold, which is hydroxylated through the action of SidB pre- or post-cyclization. Iron-dependent transporters (*sidEG*) and a suite of predicted metal-dependent transcription factors (*sidR1R2R3*) were also found within the cluster, consistent with a role in metal homeostasis. Figure 2B shows the proposed siderochelin biosynthesis pathway.

**Fig 3:**
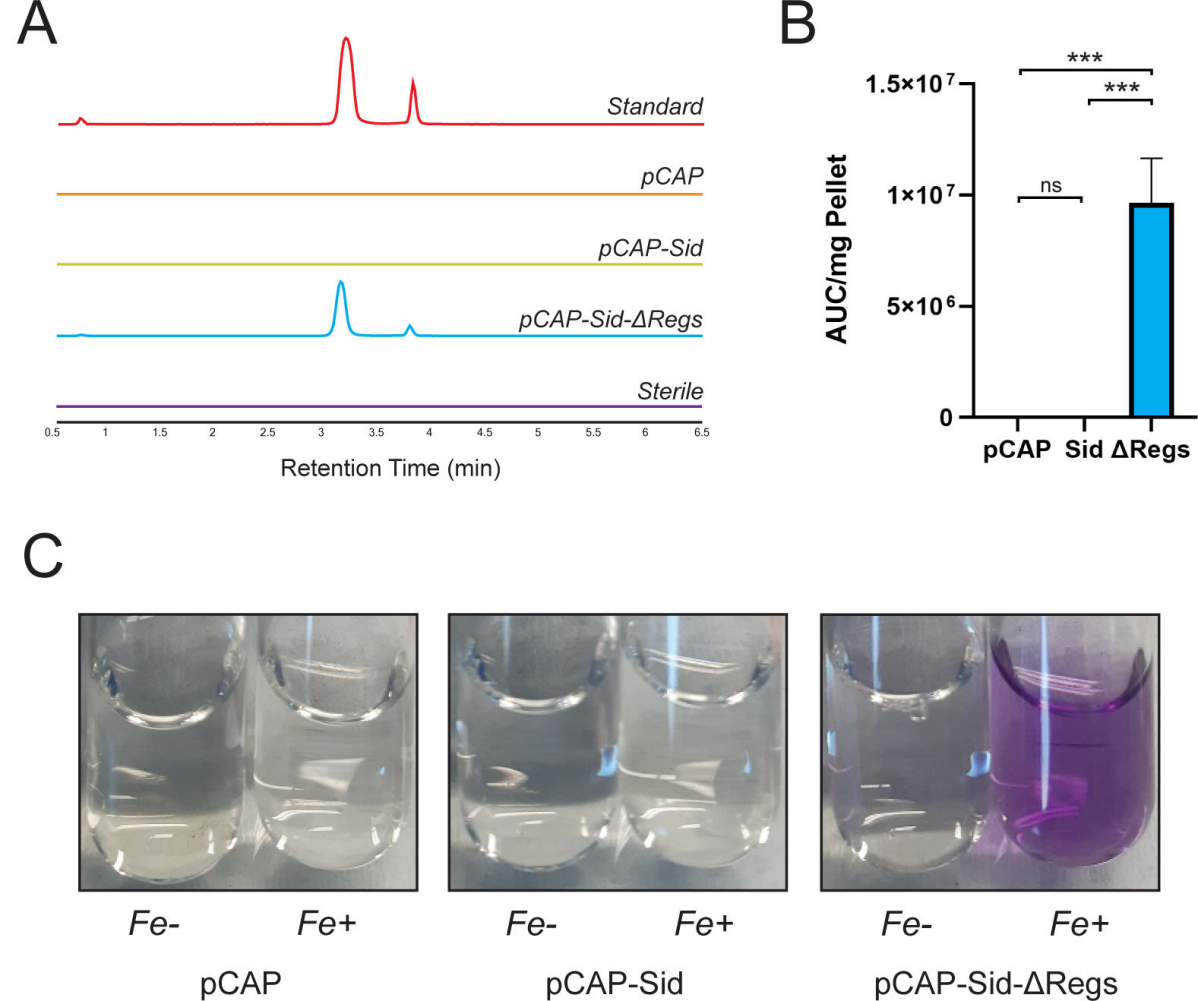
Validation of the siderochelin BGC. (**A**) Extracted ion chromatogram (EIC) traces for the m/z of siderochelin (236.1033) from strains grown in iron-depleted conditions. Traces are representative of three biological replicates. (**B**) Quantification of EIC peaks for siderochelin. Color scheme corresponds to that in panel “B.” Error bars represent the standard deviation of three biological replicates. ***: *P* ≤ 0.0001 by normal one-way ANOVA. ns: *P* ≥ 0.05. AUC: area under the curve. (**C**) Conditioned media from test strains. Fe-: grown in iron-depleted conditions; Fe+: grown in iron-replete conditions. Images are representative of three biological replicates.

To validate the role of the *sid* gene cluster in siderochelin biosynthesis, we captured the cluster of 24 predicted ORFs from WAC04611 using the capture vector pCAP03-aac (3)IV (18) and integrated it into the chassis strain *Streptomyces coelicolor* M1154 (19), creating the strain pCAP-Sid. M1154 was chosen as the chassis due to the deletion of four antibiotic BGCs and mutations in the *rpoB* and *rpsL* genes, improving metabolite production from heterologous BGCs. Surprisingly, the pCAP-Sid strain failed to produce siderochelin in minimal media, regardless of iron content (Fig. 3). Given the tight regulation of iron homeostasis in cells, we created a new construct with the three predicted transcription factors excised from the cluster (pCAP-Sid-ΔRegs). The resultant ΔRegs strain produced a robust extracted ion chromatogram (EIC) signal for siderochelin (Fig. 3B and C), demonstrating that the captured cluster is sufficient for siderochelin production. Consistent with this proposal, the conditioned media of the ΔRegs strain turns a characteristic purplish-pink color under iron-replete conditions, signaling the formation of the siderochelin-iron complex (Fig. 3D).

### SidR3 is a repressor of siderochelin production

Intrigued by the results from our ΔRegs strain, we examined the role of individual regulators included in the *sid* BGC. The cluster encodes three predicted regulators: a metal-dependent MerR family regulator (SidR1), a metal-dependent DtxR family regulator (SidR2), and a FadR-like GntR family regulator (SidR3). Although MerR family regulators are usually activators of gene transcription in the presence of their cognate metal ligand (20), DtxR family regulators are classically metal-dependent repressors of gene expression (21). GntR family regulators are typically negative regulators in the absence of their ligand (22). We hypothesized that (i) SidR1 would be a positive regulator of the *sid* BGC, increasing siderochelin levels in the presence of iron; (ii) SidR2 would be a negative regulator of the BGC, and overexpression would repress siderochelin production in an iron-dependent fashion; and (iii) SidR3 would be a negative regulator, and overexpression would repress siderochelin production, regardless of iron levels.

**Fig 4:**
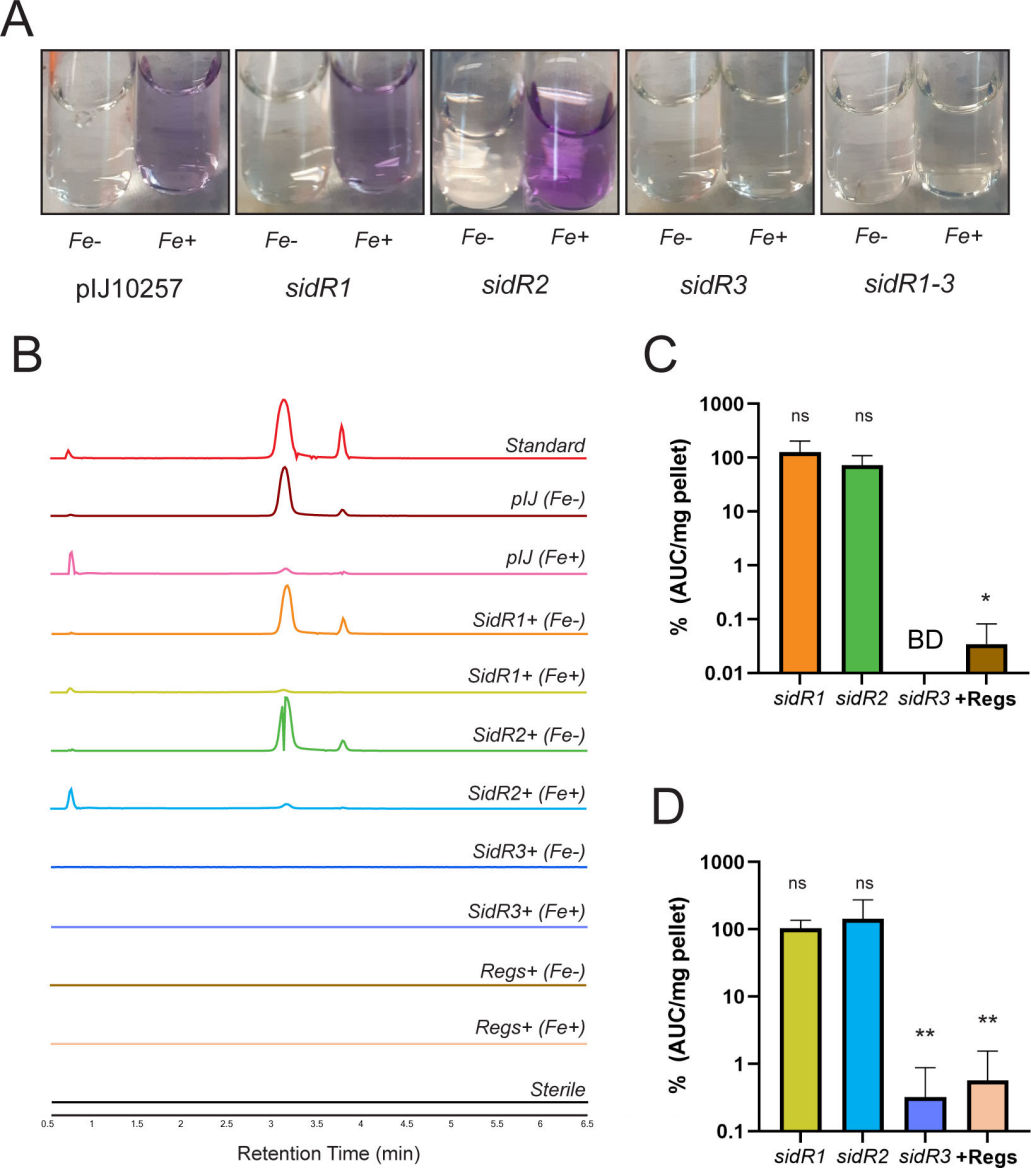
SidR3 represses siderochelin production. (**A**) Conditioned media from complement strains. Fe-: grown in iron-depleted conditions; Fe+: grown in iron-replete conditions. Images are representative of three biological replicates. (**B**) EIC traces for the m/z of siderochelin (236.1033). Traces are representative of three biological replicates. Regs+: complement of SidR1-3. pIJ: ΔRegs strain carrying empty pIJ10257 as control. (**C**) Quantification of EIC peaks for siderochelin under iron-depleted conditions and (D) iron-replete conditions. Percentages are relative to the empty pIJ10257 control. Color scheme corresponds to that in panel “B.” Statistics are relative to empty pIJ10257 control. Error bars represent the standard deviation of three biological replicates. *: *P* ≤ 0.05 by two-tailed student’s *t*-test. **: *P* ≤ 0.005. ns: *P* ≥ 0.05. BD: below the limit of detection.

To test these hypotheses, we created complementation constructs in our ΔRegs strain that placed each regulator under the control of the constitutive ermEP* promoter (23) as single genes or with all three simultaneously in their native orientation. Surprisingly, the SidR1 and SidR2 complement strains produced siderochelin levels that were comparable with the empty plasmid control, indicating no change in siderochelin production (Fig. 4); these results were reproducible, regardless of the depleted or repleted iron conditions. Examination of the EIC for the siderochelin-iron complex produced results that mirrored free siderochelin (Fig. S5), suggesting no change in siderochelin production. Sequencing of these complemented strains revealed no mutations in the complemented genes. On the other hand, complementation of SidR3 or all three regulators in their native orientation strongly repressed the levels of siderochelin, independent of iron levels (Fig. 4). Based on these data, we conclude that SidR3 is a negative regulator of the *sid* BGC.

### SidA is required for conversion of siderochelin D to siderochelin A/B

**Fig 5:**
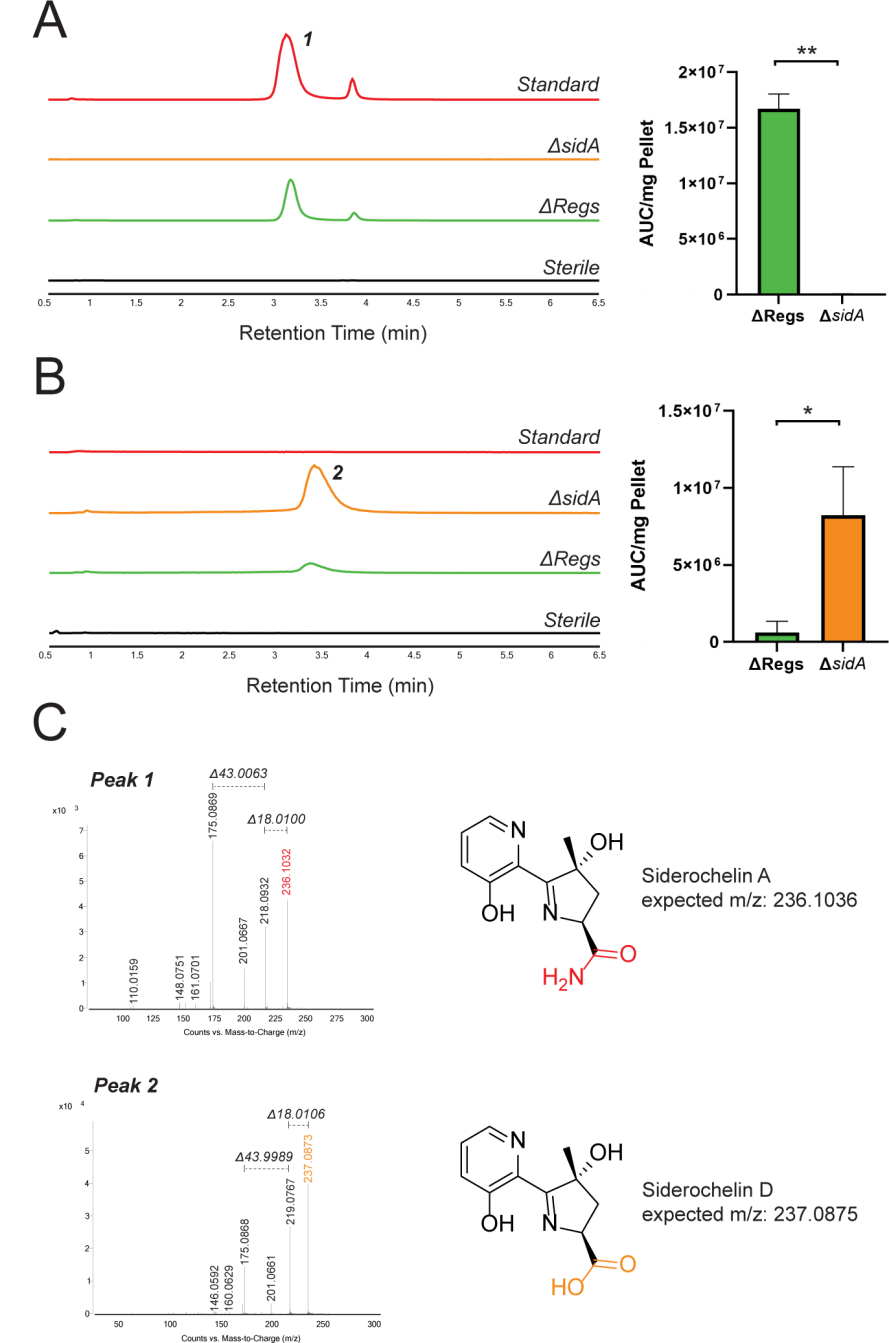
SidA converts siderochelin D to siderochelin A. (**A**) (Left) EIC traces for the m/z of siderochelin (236.1033), (Right) Quantification of EIC. **: *P* ≤ 0.005 by Student’s two-tail *t*-test. (**B**) (Left) EIC traces for the m/z of siderochelin D (237.0875). (Right) Quantification of EIC. *: *P* ≤ 0.05 by two-tailed Student’s *t*-test. Strains were grown under iron-depleted conditions. Error bars represent the standard deviation of three biological replicates. Δ*sidA*: ΔRegs strain with additional deletion of *sidA*. (**C**) MS/MS spectrum for peaks numbered as in panels “A” and “B.” Loss of 18 Da denotes loss of water; loss of 43 Da denotes loss of a carboxamide; and loss of 44 Da denotes loss of CO_2_.

The siderochelins differ from the structurally similar proferrorosamine in three aspects (Fig. 1D): (i) the phenol moiety on C4, (ii) the generation of a chiral center at C7 of the dehydropyrrole, and (iii) the presence of a carboxamide at C10 rather than a carboxylic acid. The carboxylic acid originates from the amino acid *O*-acetylserine in the proposed proferrorosamine biosynthetic pathway. Based on the presence of a serine *O*-acetyltransferase in the *sid* BGC, we propose that the siderochelins are generated similarly, and the carboxylic acid is converted to an amide after completion of the scaffold. To test this hypothesis, we scanned the *sid* BGC for candidate genes with potential amidotransferase activity. *sidA* was predicted by antiSMASH to be an asparagine synthetase (glutamine hydrolyzing), an enzyme that transfers an ammonia equivalent from glutamine to convert aspartate to asparagine. We deleted *sidA* from the ΔRegs background and tested the mutant for siderochelin production. The EIC for siderochelin was greatly diminished in the Δ*sidA* strain relative to the ΔRegs control (Fig. 5A). A new peak with an *m/z* of 237.0873 and a retention time of ~3.35 minutes was enriched in the Δ*sidA* strain (Fig. 5B). MS/MS of this molecular ion showed a fragmentation pattern similar to that of siderochelin A; the loss of 44 Da instead of 43 Da observed in siderochelin A (Fig. 5C) is consistent with the presence of a carboxylic acid instead of an amide. These data suggest that the new peak is siderochelin D (expected *m/z*: 237.0875, mass error −0.8 ppm) instead of siderochelin A and that *sidA* is required to convert the carboxylic acid of siderochelin D into the amide of siderochelin A.

### SidB is required for siderochelin production

Next, we turned our attention to the chiral center of the dihydropyrrole C7 (Fig. 1D). In the proposed proferrorosamine biosynthesis pathway, C7 originates from malonyl-CoA. In the captured *sid* BGC, *sidO* is the predicted acyltransferase of the type I PKS, and its substrate is predicted by antiSMASH to be methylmalonyl-CoA rather than malonyl-CoA, consistent with the presence of the methyl group on C7. On the other hand, the source of the hydroxyl is not obvious. We chose to investigate *sidB*, a gene predicted to encode a cupin domain-containing protein. The cupin domain-containing proteins are a diverse superfamily with enzymatic and nonenzymatic functions (24). The largest subgroup of enzymes within this family, the 2-oxoglutarate/Fe^2+^-dependent oxygenases, catalyze a variety of reactions such as hydroxylation, epoxidation, and halogenation, which would be consistent with installation of the dihydropyrrole hydroxyl. These enzymes share a conserved HX(D/E)…H triad within the cupin domain, which coordinates the metal cofactor (25). If SidB is the hydroxylase, it should also possess this conserved triad. We created a model of SidB using AlphaFold and then aligned the cupin barrel onto that of prolyl hydroxylase from *B. anthracis* (PDB 5HV4) (Fig. S6) (26, 27). From the protein sequence alignment, SidB appears to have a rearranged triad (HXH…E); nevertheless, the model predicts the presence of the triad in a correct 3D arrangement, which should still be conducive to metal coordination.

**Fig 6:**
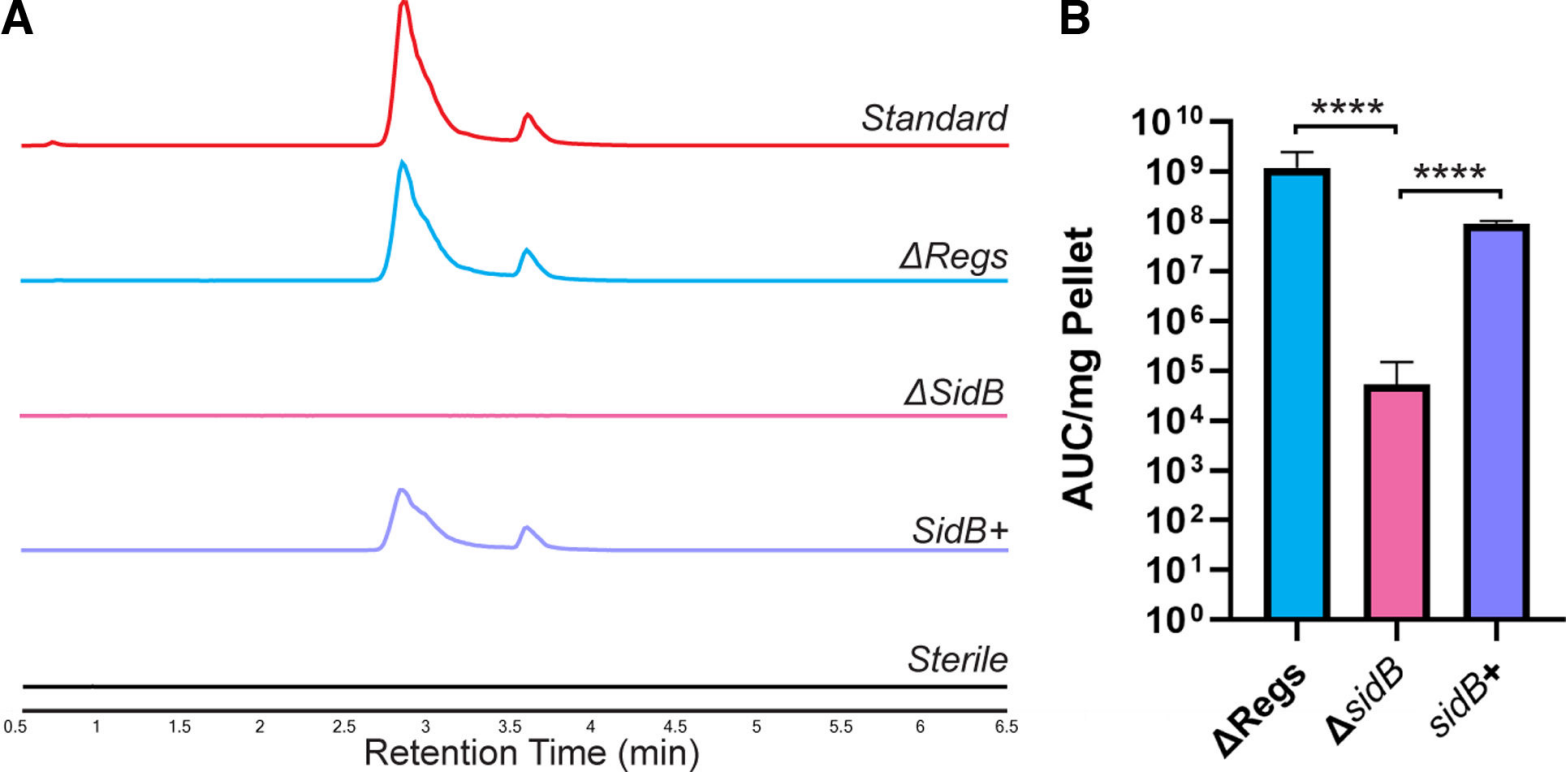
*sidB* is required for siderochelin production. (A) EIC traces for the m/z of siderochelin (236.1033) from strains grown in iron-depleted conditions. Traces are representative of three biological replicates. *sidB+*: Δ*sidB* strain complemented with pIJ10257-*sidB* construct. ΔRegs and Δ*sidB* strains carry empty pIJ10257 as a control. (B) Quantification of EIC traces. Error bars represent the standard deviation of three biological replicates. ****: *P* ≤ 0.0001 by two-tailed Student’s *t*-test.

Based on the model, we hypothesized that *sidB* encodes a 2-oxoglutarate/Fe^2+^-dependent oxygenase that hydroxylates siderochelin. We deleted *sidB* from the ΔRegs background and monitored siderochelin production. If SidB is the hydroxylase, we would predict the appearance of an *m/z* of 220.1036 (dehydroxylated siderochelin) in its absence. Although we did observe a near complete loss of siderochelin production (Fig. 6), we failed to see the appearance of a peak consistent with the presence of dehydroxylated siderochelin or another reasonable intermediate in the conditioned media or cell pellet extracts. Complementation of *sidB* was able to partially rescue siderochelin production (Fig. 6). Although our data do not conclusively rule out SidB’s role as the siderochelin hydroxylase, it does demonstrate that SidB is crucial for siderochelin production.

## DISCUSSION

Bacteria compete with their neighbors for access to scarce resources (28), and the production of secondary metabolites such as siderophores grants them a competitive advantage over their rivals (3). Microbial genome sequencing has revealed the tremendous potential for secondary metabolite production among actinobacteria (2, 29). The genus *Streptomyces* is particularly more prolific than previously thought, based solely on historical laboratory fermentation studies (29). Nevertheless, most secondary metabolite BGCs remain silent under laboratory conditions, and their products remain unknown (30–32). Here, we utilize coculture as a mimic for natural bacterial competition to stimulate secondary metabolite production, resulting in the identification of the siderochelin BGC.

Only nine molecular masses were consistently enriched more than 10-fold (relative to each constituent strain alone) across three biological trials. Two of these nine masses were identified as siderophores desferrioxamine B and siderochelin A/B. The low hit rate is likely due to our high fold cutoff for hit calling and considerable heterogeneity among biological replicates (Fig. S1). Since siderophores are among the most overproduced secondary metabolites in these studies, they will likely be consistently rediscovered in future coculture screens. Adding extra iron to the coculture media may help suppress siderophore production and minimize the rediscovery of known siderophores.

The *Tsukamurella* strain WAC06889b is well represented as a constituent strain among the hits, particularly when paired with the *Amycolatopsis* strain WAC04611. *Tsukamurella* and other mycolic acid-containing bacteria have been shown to induce red pigment in *S. lividans* TK23 and other *Streptomyces* species under both solid media and broth conditions (33). Intriguingly, the WAC06889b and WAC04611 coculture only increased siderochelin production when in direct contact, and efforts to induce production in broth proved unsuccessful. Additionally, neither of the other mycolic acid-containing bacteria in our coculture screen (*Nocardia* sp. WAC07162 and *Nocardia* sp. WAC06296) successfully induced siderochelin production when cocultured with WAC04611. The precise nature of the signaling between WAC06889b and WAC04611 required for siderochelin production remains to be further elucidated.

Since siderochelin is a siderophore, we expected to observe the overproduction of siderochelin in iron-depleted conditions. However, neither our pCAP-Sid heterologous expression strain (Fig. 3) nor WAC04611 produced siderochelin under iron-depleted conditions after 9 days of growth; WAC04611 required the presence of WAC06889b, and our heterologous expression strain required the removal of at least *sidR3* (Fig. 3 and 4). This suggests that additional signals beyond iron levels alone are necessary for siderochelin production.

Although the siderochelins were first discovered in 1981, the BGC responsible for their production remained unknown (11). We used the BGC and proposed biosynthesis of proferrorosamine (16) to identify the siderochelin BGC in WAC04611. Although the siderochelins and proferrorosamine are structurally very similar, there is minimal nucleotide identity and synteny shared between their BGCs; only *sidD* and *sidN* show significant nucleotide identity to their counterparts in the proferrorosamine BGC (*rosC* and *rosA*, respectively) (Fig. S7). This observation suggests that it is the biosynthetic logic that is similar between the two BGCs. Nevertheless, this conserved biosynthetic logic was sufficient to guide us to the siderochelin BGC.

The captured cluster is a ~34 kb length of DNA containing 24 ORFs. Although further genetic confirmation is needed, we speculate that the BGC boundaries are *sidR1* and *sidO* (Fig. 2). Between *sidR1* and *sidO* are ORFs predicted to build the siderochelin scaffold, based on the model for proferrorosamine production: 3HPA biosynthesis, type 1 PKS machinery for linking 3HPA and methylmalonyl-CoA, *O*-acetylserine production, and a *rosC* homolog (*sidD*). Curiously, unlike in the proferrorosamine PKS module RosF, the domains of the siderochelin PKS are predicted to be discrete proteins rather than a single module (Fig. 2A; Fig. S4); the ketosynthatse SidK (or SidL) acyltransferase SidO, and acyl carrier protein SidM would need to form a complex to complete the module. We propose the following model in Fig. S8: In a fashion similar to MbtA from the mycobactin BGC (34), the AMP-dependent synthetase and ligase SidN activates 3-HPA and loads the resulting 3-HPA-CoA onto a free-standing SidM. Loaded SidM then passes its cargo to SidK(L) in the SidK(L)-SidO-SidM complex. SidM of the SidK(L)-SidO-SidM complex is subsequently loaded with methylmalonyl-CoA by SidO and linked to 3-HPA. An unknown thioesterase then releases the product for further processing. Overall, each protein’s exact role(s) in this model remains to be determined.

3HPA is a building block for secondary metabolites such as pristinamycin (35), virginiamycin S (35), and viridogrisein (36) (synthesized by the *sna*, *vir*, and *sgv* BGCs, respectively). Biosynthesis of 3HPA requires a suite of four enzymes: an L-lysine-2-aminotransferase, an FAD-dependent dehydrogenase, and a two-component monooxygenase system consisting of a flavin mononucleotide (FMN)-dependent monooxygenase and an NADH-dependent FMN reductase (37). The *sidH*, *sidI*, and *sidJ* protein products are similar to those from the *sgv* BGC (58% ID to SgvL, 61% ID to SgvF, and 57% ID to SgvS, respectively). However, the NADH-dependent FMN reductase appears absent from the siderochelin BGC. It may be one of the ORFs of unknown function within the BGC or a promiscuous exogenous gene within the chassis strain fills the role. Further work is needed to identify the missing FMN reductase.

Once transferred to the chassis strain, the captured BGC failed to produce siderochelin until the three predicted regulators, SidR1-SidR3*,* were deleted (Fig. 3). SidR1 and SidR2 are predicted to be metal-dependent regulators, with SidR2 specifically being a predicted DtxR homolog. The DtxR family of regulators includes negative regulators that bind to the consensus sequence 5′-TTAGGTTAGCCTAACCTAA-3′ in the presence of excess iron to repress gene transcription (38). Initial scans of the siderochelin BGC intergenic regions identified three potential DtxR binding sites with high sequence identity to the consensus sequence (Fig. S9). Surprisingly, complementing SidR2 in our ΔRegs mutant did not affect siderochelin production levels, regardless of the presence of iron (Fig. 4). DtxR homologs are common among GC-rich Gram-positive bacteria, including our chassis strain, which possesses two homologs (DmdR1 and DmdR2) (39); DmdR1 and/or DmdR2 may cross-regulate the exogenous siderochelin BGC and mask any SidR2 complementation phenotype. Alternatively, although we sequence-validated the chromosomally integrated *sidR2* complement, efficient protein expression remains to be validated.

Like SidR2, complementation of the predicted MerR family regulator SidR1 in our ΔRegs mutant did not affect siderochelin production, regardless of the presence of iron. Members of the MerR family are predominantly transcription activators, activated by binding a small molecule ligand or metal; the activated transcription factor binds to a dyad inverted repeat within the target promoter, aligning the −35 and −10 sequences to promote binding of RNA polymerase (20). A preliminary screen of the siderochelin BGC’s intergenic regions identified a single perfect dyad inverted repeat upstream of *sidR1-R3* (Fig. S9). Based on this observation, we speculate that SidR1 may only autoregulate itself and the downstream regulators to fine-tune BGC gene expression. It is also possible that SidR1 may be required to regulate the two other predicted siderophore BGCs in WAC04611, neither of which possesses any predicted metal-dependent regulators of their own. Alternatively, SidR1 may not respond to iron at all; to date, MerR family regulators have been identified that sense Hg^2+^, Zn^2+^, Co^2+^, Pb^2+^, Cd^2+^, Cu^1+^, Au^1+^, and Ag^1+^, but none that bind iron have been discovered (20). SidR1 may sense one of these other metals and regulate operons outside the siderochelin BGC. Further study is needed to dissect SidR1’s full role in siderochelin biosynthesis.

Complementation of the GntR family regulator SidR3 resulted in strong repression of siderochelin production in our ΔRegs mutant (Fig. 4). SidR3 is predicted by the conserved domain database (40) to belong to the FadR subfamily of GntR regulators. FadR family members typically bind to a palindromic consensus sequence of N_y_GTM-N_0-1_-KACN_y_, repressing gene expression and dissociating only upon binding of a small molecule ligand (typically a fatty acyl-CoA, sugar, or other catabolic substrate) (22, 41). A pair of sequences matching the FadR binding consensus sequence was identified in the intergenic region upstream of *sidH* and other genes predicted to be involved in the early steps of siderochelin biosynthesis (Fig. S9). SidR3 may regulate the early steps of siderochelin production by sensing levels of the required substrates. SidR3’s exact promoter binding sites and ligand(s) remain to be determined.

Siderochelin A and siderochelin D differ only by the presence of an exocyclic carboxamide or carboxylic acid, respectively, on their C10 carbon (Fig. 5). The model of proferrorosamine biosynthesis proposed by Born et al. suggests that the carboxylic acid derives from the incorporation of *O*-acetylserine into the dihydropyrrole ring (16). If this also holds for siderochelin biosynthesis, the C10 carboxyl group must be modified to an amide. Our experiments with *sidA* support this hypothesis as deletion of *sidA* leads to the loss of the *m/z* for siderochelin A and the accumulation of the *m/z* for siderochelin D. This suggests that siderochelin D is likely an intermediate of siderochelin A production. However, the biological importance of this modification is unknown; proferrorosamine lacks the amide group but can still chelate Fe^2+^, suggesting the exact identity of this substituent is not crucial for its iron scavenging ability.

Unlike proferrorosamine, the siderochelins possess a hydroxyl group at the C7 position. Although the timing of this hydroxylation cannot be pinpointed at this time, we speculate, based on the structure of siderochelin D, that it occurs either before the incorporation of *O*-acetylserine or just after but before modification by SidA. We hypothesized that *sidB* was the hydroxylase responsible due to its homology to cupin domain-containing proteins, specifically 2-oxoglutarate/Fe^2+^-dependent oxygenases. Additionally, the dihydropyrrole ring that is hydroxylated bears a striking resemblance to proline, an amino acid that is frequently hydroxylated by 2-oxoglutarate/Fe^2+^-dependent oxygenases (25, 42, 43). However, although deletion of *sidB* did lead to a loss of siderochelin production, we were unable to identify a predicted intermediate mass (Fig. 6). Loss of the hydroxyl group may destabilize the molecule, or perhaps, a downstream enzyme such as SidD requires it for substrate recognition (if hydroxylation occurs before *O*-acetylserine incorporation).

Many silent BCGs are waiting to be activated and characterized. Coculture and other growth conditions that more closely mimic natural environments are an increasingly popular strategy to induce silent BGCs. When used to complement synthetic biology/metabolomics, genetic engineering, and chemical inducers (4), these strategies promise to further aid the discovery of novel secondary metabolites for pharmaceuticals and beyond.

## MATERIALS AND METHODS

### Strains, growth conditions, and reagents

All strains used in this study are listed in Table S1. *E. coli* strains were routinely grown on LB agar at 37°C or in LB broth at 37°C shaken at 250 rpm, supplemented with 50 µg/mL kanamycin,100 µg/mL ampicillin, 50 µg/mL apramycin, 5 µg/mL chloramphenicol, or 150 µg/mL hygromycin when required. WAC library strains were routinely grown on Bennett’s agar (10 g/L potato starch, 2 g/L casamino acids, 1.8 g/L yeast extract, 2 mL/L Czapek mineral solution [100 g/L KCl, 100 g/L MgSO_4_·7H_2_O, 120 g/L NaNO_3_, 2 g/L FeSO_4_·7H_2_O, 2 mL/L concentrated HCl], and 15 g/L agar; pH-adjusted to 6.8) at 30°C or in *Streptomyces* antibiotic production media (SAM) (15 g/L glucose, 15 g/L soya peptone, 5 g/L NaCl, 1 g/L yeast extract, 1 g/L CaCO_3_, 2.5 mL/L glycerol; pH-adjusted to 6.8) at 30°C, 250 rpm. *Streptomyces coelicolor* M1154 and its derivatives were routinely grown on soy flour-mannitol (SFM) agar (20 g/L soy milk powder, 20 g/L D-mannitol, and 15 g/L agar; pH-adjusted to 7.2–7.4) supplemented with 20 mM MgCl_2_ and 50 µg/mL kanamycin, 50 µg/mL apramycin, 50 µg/mL hygromycin, or 25 µg/mL nalidixic acid when required.

For siderochelin production experiments, all strains were grown in liquid minimal media at 30°C, 250 rpm. Briefly, base minimal media (0.5 g/L L-asparagine, 0.5 g/L K_2_HPO_4_; pH 7.0–7.2) flowed through a gravity Chelex 100 (Bio-Rad) column per manufacturer’s instructions. After autoclaving, glucose and MgSO_4_∙7H_2_O were added to a final concentration of 10 g/L and 0.2 g/L, respectively. For iron-replete conditions, 4 mM FeSO_4_:400 mM NaC_6_H_5_O_7_ (pH 7.0) solution was added to a final concentration of 36 µM FeSO_4_. An equivalent amount of 400 mM NaC_6_H_5_O_7_ (pH 7.0) was added for iron-depleted conditions.

All plasmids used in this study are listed in Table S1. All primers and gBlocks used in this study were purchased from IDT Inc. and are listed in Table S2.

### Mass spectrometry

High-resolution mass spectrometry (HR-MS) of coculture and siderochelin expression experiments were recorded on an Agilent 6550 iFunnel Q-TOF mass spectrometer equipped with an inline Agilent 1290 high-performance liquid chromatography (HPLC) system using electrospray ionization in a positive mode. Samples were fractionated on a reverse-phase C8 analytical column (Agilent Eclipse XDB-C8, 3.5 µm pore size, 2.1 × 100 mm column) with a flow rate of 0.4 mL/minute. Temperature was held constant at 45°C. The mobile phase was water + 0.1% formic acid (solvent A) and acetonitrile + 0.1% formic acid (solvent B). Solvent B was held constant at 5% for 1 minute and then increased over 6 minutes to 97%; this was held constant for 1 minute and then decreased over 0.5 minutes to 5% and held constant for an additional 0.5 minutes.

### Coculture experiments

WAC strains were grown in 4 mL SAM cultures at 30°C, 250 rpm for 4 days. Cells were rinsed twice with sterile saline and then resuspended to a concentration of 4 × 10^7^ spores/mL (or CFU/mL for non-sporulating strains). 2.5 µL of cell suspensions were added to 1 mL of Bennett’s agar in 24 well plates either at opposing sides of the well or mixed and placed at the well’s center. Plates were incubated at 30°C for 9 days. Individual wells were then harvested and extracted in 1 mL of 1:1 methanol:butanol overnight at 4°C. Extracts were filtered through a 0.2 µm filter and stored at −80°C until needed. Coculture experiments were performed in biological triplicate.

### Metabolomics

One microliter of undiluted coculture extracts was injected in duplicate for HPLC-MS (see mass spectrometry methods above). Duplicate chromatograms were imported into the MassHunter Profinder software (Agilent Technologies, Inc.), and respective duplicate chromatograms were grouped together. Chromatograms were time-aligned to the chromatogram of a mixture containing 2 µL of each coculture extract followed by entity extraction (recursive extraction for small molecules/peptides, default settings). Extracted entities were exported to a Profinder archive file. The Profinder archive file was then imported into the MassHunter Profiler Professional software (Agilent Technologies, Inc.). Coculture samples were compared with the samples of their constituent strains using default settings except for the following: the minimum peak cutoff was set to 1 × 10^5^, and the MFE setting was set to 85. Entities that were upregulated ≥10-fold relative to either constituent strain alone (*P*-value ≤ 0.05) were considered hits.

### Purification of siderochelins from WAC04611-WAC06889b cocultures

WAC04611 and WAC06889b monocultures were grown for 4 days in SAM medium as described above. Both strains were rinsed twice in sterile saline and then resuspended to a final OD_600_ of 0.43. The strains were mixed and plated in 5 µL spots across Bennett’s agar with a multichannel pipette. Plates were incubated at 30°C for 9 days and then extracted overnight in water. Crude extracts were mixed 1:1 with acetonitrile, and the precipitate was collected by centrifugation and discarded. The supernatant was dried by lyophilization and resuspended in water + 1 mM FeSO_4_.

The resuspended sample was fractionated on a gravity LH-20 size-exclusion chromatography (3.66 × 43.5 cm). The mobile phase was room-temperature 1:1 H_2_O:acetonitrile with a 2.25 mL/minute flow rate. Two hundred milliliters of solvent were allowed to elute before the collection of 184 mL fractions. Fractions with pink coloration were pooled. Samples of the remaining fractions were tested by the addition of 1 mM FeSO_4_; fractions with a visible color change were pooled together. Pooled fractions were dried by lyophilization and resuspended in H_2_O.

Pooled fractions were fractionated into 12 mL fractions by reverse-phase liquid chromatography using a 86g C18 column (BÜCHI Labortechnik) on a CombiFlash system (Teledyne Technologies, Inc.) with a flow rate of 30 mL/minute. The mobile phase was 5 mM ammonium acetate pH 5 (solvent A) and acetonitrile (solvent B). Solvent B was held constant at 5% for 5 minutes and then increased over 5 minutes to 10% solvent B and held constant for an additional 5 minutes. Solvent B was then increased over 3 minutes to 80% and held constant for 3 minutes. Finally, solvent B was decreased over 3 minutes to 5% and held at 5% for 3 minutes. Pink fractions (fractions 9–13) were pooled, whereas all other fractions were sampled and tested with 1 mM FeSO_4_ as previously stated. Fractions with notable color change (fractions 20–21) were pooled together. Pooled fractions were dried by lyophilization and resuspended in H_2_O.

Pooled combiflash fractions 20–21 were further purified on a reverse-phase C8 semiprep HPLC column (Eclipse XDB-C8, 5 µm pore size, 9.4 × 250 mm column) with a flow rate of 3 mL/minute. Temperature was held constant at 30°C. The mobile phase was water + 0.1% formic acid (solvent A) and acetonitrile + 0.1% formic acid (solvent B). Solvent B was held constant at 5% for 1 minute and then increased over 19 minutes to 20%; it was further increased over 7 minutes to 95% and held constant for 3 minutes. Finally, solvent B was decreased over 5 minutes to 5% and held constant for 3 minutes. Peaks were collected manually following UV 550 nm and UV 325 nm. The peak collected at 18.5–19.0 minutes was found by HPLC-MS to contain the target compound.

Pooled combiflash fractions 9–13 were fractionated on the C8 column in the same fashion as fractions 20–21 above with the following modification. To dissociate the siderophore-iron complex, pooled fractions were incubated in 5% formic acid for 1 h before HPLC. The peak collected at 18.5–19.0 minutes was pooled with the corresponding peak from fractions 20–21. The peak collected at 8.5–9.0 minutes (UV 550, pink coloration) was iteratively dried down by lyophilization, resuspended in 5% formic acid, and reinjected onto the HPLC until dissociation of the chelated iron complex was complete.

Samples containing the 18.5–19.0 C8 peak were pooled, dried by lyophilization, and then fractionated on a reverse-phase C4 prep HPLC column (XBridge Protein BEH C4, 5 µm pore size, 10 × 250 mm column) with a flow rate of 3 mL/minute. Temperature was held constant at 30°C. The mobile phase was water + 0.1% formic acid (solvent A) and acetonitrile + 0.1% formic acid (solvent B). Solvent B was held constant at 5% for 1 minute and then increased over 14 minutes to 27.1%; it was further increased over 5 minutes to 95% and held constant for 5 minutes. Finally, solvent B was decreased over 5 minutes to 5% and held constant for 3 minutes. Peaks were collected manually following UV 550 nm and UV 325 nm. The peak collected at 11.5–12.5 minutes was found by HPLC-MS to contain siderochelin.

### NMR spectroscopy

The 1D and 2D NMR experiments were performed using Bruker AVIII 700 MHz instrument equipped with a cryoprobe in deuterated DMSO. Chemical shifts are reported in parts per million relative to tetramethyl silane using the residual solvent signals at 2.50 ppm in proton NMR and 39.5ppm in carbon NMR as an internal signal.

### Genome sequencing

Genomic DNA extraction and Illumina sequencing of WAC library strains were carried out as previously described (44). gDNA from strains was prepared for Illumina Sequencing (MiSeq 2 × 250 bp reads) using the NEB Next Ultra II kit (New England Biosciences) with 444 ng input DNA (sonicated to 600 bp via Covaris MicroTUBE) and a double size selection with purification beads. Sequencing was performed by the McMaster Genomics Facility in the Farncombe Institute at McMaster University (Hamilton, ON, Canada). Sequencing reads were trimmed using skewer v0.2.2 (-q 25 and -Q25;) (45) and merged using FLASH v1.2.11 with default parameters (46).

High molecular weight gDNA of strains was isolated using the salting out procedure (47) followed by removing the RNA by RNase A treatment. For Nanopore sequencing of WAC strains, 450 ng of high-molecular weight gDNA was prepared using the Rapid Barcoding Kit (SQK-RBK004) from Oxford Nanopore Technologies. This sample was pooled at equal volumes with four other genomes and sequenced on a MinION R9.4.1 flow cell for 48 h. Read traces were classified using Deepbinner v0.2.0 (48) before basecalling with ONT’s Guppy basecaller (2.3.1; dna_r9.4.1_450bps configuration). Reads were binned using Deepbinner v0.2.0 (48) and then trimmed using Porechop v0.2.4 (https://github.com/rrwick/Porechop). Trimmed and merged Illumina reads from previous Illumina sequencing, along with trimmed long reads, were *de novo* assembled using Unicycler v0.4.8b (49) using Pilon v1.23 (50) and SPAdes v3.13.0 (51). The *sid* biosynthetic gene cluster was submitted to GenBank (accession number PP928996).

### TAR cloning of siderochelin BGC

TAR cloning was performed by following the standard protocol (52). Synthesized sid-gBlock targeting siderochelin BGC as shown in Table S2 was inserted into the TAR cloning vector pCAP03-aac (3)IV between *Xho*I/*Nde*I sites through Gibson assembly, resulting in the capture vector pCAP03-SidCap. The capture vector was linearized by *Pme*I digestion, purified by PCR clean-up kit (GeneJET, Thermo Fisher), and transformed into yeast spheroplast cells. gDNA of WAC04611 was isolated using the salting out procedure (47) followed by RNA removal by RNase A treatment. Purified high-molecular weight gDNA was then digested with *Afl*II and *Bsu*36I to release the siderochelin BGC and purified through sodium acetate precipitation. Linearized pCAP03-SidCap capture plasmid (~500 ng) and digested gDNA (~2 µg) were mixed and co-transformed into *S. cerevisiae* VL6-48N spheroplast cells, plated onto SD-Trp + 5 FOA selection medium, and grown for 3–5 days. Yeast transformants were picked into liquid SD-Trp medium, grown for 24 h, and then, the plasmid DNA was extracted using the alkaline lysis method for PCR screening. Positive hits were selected and re-transformed into *E. coli* Top10 cells through electroporation, followed by confirmation using restriction digestion mapping. The confirmed construct was called pCAP03-Sid and conjugated into *S. coelicolor* M1154 (see below).

### *E. coli-Streptomyces* conjugations

Desired constructs were transformed into *E. coli* ET12567/pUZ8002-neo::bla *via* electroporation and shuttled into *S. coelicolor* M1154 by *E. coli-Streptomyces* interspecies mating. *E. coli* ET12567/pUZ8002-neo::bla containing desired constructs were grown overnight at 37°C, 250 rpm in LB supplemented with required antibiotic combinations. 0.5 mL of cells were harvested by centrifugation and rinsed twice with 1 mL fresh LB followed by resuspension in 0.1 mL LB. Fifty microliters of *S. coelicolor* M1154 spores were thawed from frozen stocks, rinsed twice in 1 mL fresh LB, then resuspended in 500 µL 2 x YT medium and heat-activated for 10 minutes at 50°C. *E. coli* and heat-activated *Streptomyces* spores were mixed and plated onto SFM + 20 mM MgCl_2_ agar plates. Plates were incubated at 30°C overnight. Nalidixic acid and construct-specific antibiotics were combined in 1 mL ddH_2_O and overlaid onto the conjugation plates (for selection concentrations, see *Strains, Growth Conditions, and Reagents* section). Conjugation plates were then incubated at 30°C for a further 3–5 days. Antibiotic-resistant exconjugants were confirmed by PCR and used to prepare seed cultures for heterologous siderochelin expression.

### Gene deletion in pCAP-Sid

Deletion of transcriptional regulators in the pCAP-Sid construct was performed using λ-RED recombineering technology (53). Briefly, primers AJS50/AJS51 were used to amplify the *aac* (3*)IV* resistance cassette from pCAP03-aac (3)IV *via* two-step PCR. The amplicon was gel-purified by a gel extraction kit (GeneJET, Thermo Fisher) and electroporated into *E. coli* BW25113/pKD46/pCAP-Sid competent cells, resulting in pCAP-Sid-ΔRegs::aac (3)IV. The construct was digested with *Nsi*I and the plasmid backbone self-ligated with T4 DNA ligase (Thermo Fisher) to excise the *aac (3)IV* cassette, resulting in pCAP-Sid-ΔRegs; excision was validated by whole plasmid sequencing (Plasmidsaurus). Deletion of *sidA* or *sidB* from the pCAP-Sid-ΔRegs construct was performed in a similar manner using primers AJS56/AJS57 and AJS75/AJS76, respectively; verified constructs were called pCAP-Δ*sidA*::aac (3)IV and pCAP-Δ*sidB*::aac (3)IV. Constructs were conjugated into *S. coelicolor* M1154.

### Complementation of gene deletions

Targeted genes were amplified *via* two-step PCR. The amplicons were inserted into pIJ10257 between the *Nde*I/*Hind*III sites downstream of the ermEp* promoter *via* Gibson assembly. Constructs were validated by Sanger sequencing using primers AJS67/AJS68. Constructs were then conjugated into *S. coelicolor* M1154 alongside their respective gene deletion constructs.

### Heterologous production of siderochelin

Seed cultures of *S. coelicolor* M1154 strains were grown for 4 days in 3 mL SAM medium with required antibiotic selection at 30°C, 250 rpm. Cultures were rinsed twice in sterile saline and then inoculated to a final OD_600_ of 0.43 in 4 mL of iron-depleted minimal medium +/− exogenous iron. Cultures were incubated in plastic test tubes with a single glass bead at 30°C, 250 rpm for 10 days. The conditioned media was harvested and dried by Genevac centrifugal evaporator (SP Scientific) and resuspended in 0.4 mL ddH_2_O. 1 µL of resuspended extracts (diluted 1:5 in H_2_O) was analyzed by HPLC-MS.

## SUPPLEMENTAL MATERIAL

Supplemental material mbio.01871-24-s0001.docx Supplemental tables and figures.

ASM does not own the copyrights to Supplemental Material that may be linked to, or accessed through, an article. The authors have granted ASM a non-exclusive, world-wide license to publish the Supplemental Material files. Please contact the corresponding author directly for reuse.
