# Supplementary material for: Identification and characterization of the siderochelin biosynthetic gene cluster via coculture
Source: mBio. 2024 Aug 27;15(10):e01871-24. doi: 10.1128/mbio.01871-24 (PMC11481915; doi:10.1128/mbio.01871-24)
Supplement: Supplemental material — Supplemental tables and figures. [file mbio.01871-24-s0001.docx]

**Supplementary Table S1: Strains and plasmids used in this study**

| **Strain** | **Description** | **Reference** |
| --- | --- | --- |
| *E. coli* Top10 | General cloning and plasmid maintenance | Invitrogen |
| *E. coli* BW25113 Δ*bamB*Δ*tolC* | Outer membrane-weakened strain | (1) |
| *E. coli* ET12567/puZ8002-neo::bla | *E. coli-Streptomyces* mating strain; AMP^R^, CAM^R^ | Lab stock |
| *E. coli* BW25113/pKD46 | λ-RED PCR targeting host strain | (2) |
| *S. cerevisiae* VL6-48N | *MAT α, his3-D200, trp1-Δ1, ura3-Δ1, lys2, ade2−101, met14, psi+cirO*, TAR host strain | (3) |
| *S. coelicolor* M1154 | Δ*act* Δ*red* Δ*cpk* Δ*cda* *rpoB*[C1298T] rpsL[A262G] | (4) |
| *Amycolatopsis sp.* WAC04611 | Siderochelin producer; coculture strain | Lab stock |
| *Nocardia sp.* WAC06296 | Coculture strain | Lab stock |
| *Tsukamurella sp.* WAC06889b | Coculture strain | Lab stock |
| *Nocardioides sp.* WAC07034 | Coculture strain | Lab stock |
| *Streptomyces sp.* WAC07090 | Coculture strain | Lab stock |
| *Streptomyces sp.* WAC07091 | Coculture strain | Lab stock |
| *Streptomyces sp.* WAC07154 | Coculture strain | Lab stock |
| *Streptomyces sp.* WAC07158 | Coculture strain | Lab stock |
| *Nocardia sp.* WAC07162 | Coculture strain | Lab stock |
| **Plasmids** |  |  |
| pCAP03-aac(3)IV | *ura3 CEN/ARS Trp1 ori tarJ-oriT attP-int*_ΦC31_ *neo aac(3)IV*, Tar cloning capture vector | (5) |
| pCAP03-Sid | pCAP03-aac(3)IV bearing Sid BGC; KAN^R^ | This work |
| pCAP03-Sid-ΔRegs | pCAP03-Sid with regulators deleted; KAN^R^ | This work |
| pCAP03-Δ*sidA*::aac(3)IV | pCAP03-Sid-ΔRegs with *sidA* interrupted by aac(3)IV; APRA^R^, KAN^R^ | This work |
| pCAP03-Δ*sidB*::aac(3)IV | pCAP03-Sid-ΔRegs with *sidB* interrupted by aac(3)IV; APRA^R^, KAN^R^ | This work |
| pIJ10257 | *hph ermEp* traJ-oriT attp-int_ΦBT1_*, *Streptomyces* gene over-expression under the control of ermEp* strong promoter; HYG^R^ | (6) |
| pIJ10257-*sidR1* | *sidR1* overexpression construct on pIJ10257; HYG^R^ | This work |
|  |  |  |
| pIJ10257-*sidR2* | *sidR2* overexpression construct on pIJ10257; HYG^R^ | This work |
| pIJ10257-*sidR3* | *sidrR3* overexpression construct on pIJ10257; HYG^R^ | This work |
| pIJ10257-Regs | *sidR1*-*sidR2*-*sidR3* overexpression construct on pIJ10257; HYG^R^ | This work |
| pIJ10257-*sidB* | *sidB* overexpression construct on pIJ10257; HYG^R^ | This work |
|  |  |  |

**Supplementary Table S2: gBlock and primers used in this study**

| **Name** | **Sequence (5’-3’)** | **Description** |
| --- | --- | --- |
| pCAP-SidCap | ggtataaatagtggctcgagCAGCCAACACAAGTACGCCCAAGTACCCGAAGTAACGAACGAAACAGGAAgtttaaacAACCAGCCAAGCGTCGAAGAAAGAAGCAAGCGAACGCCCGGCAAAGCGTTcatatgtcgaaagctacata | TAR cloning targeting construct;  pCAP03-aac(3)IV gibson overhangs  Sid BGC upstream hook  Sid BGC downstream hook |
| Primers |  |  |
| AJS37 | TTCCAAGCATCGATCCAG | Diagnostic primer for capture of Sid BGC |
| AJS38 | TCGGTTTCTTCGGTGATC | Diagnostic primer for capture of Sid BGC |
| AJS50 | atcctcaacctgggttgagttcaaatacgttcatggaatccgcatcgatcatgcatTGCAGCTCACGGTAACTGAT | Gibson primer for Sid regulator KO insert |
| AJS51 | ggccgtccgtacgcctcggctgagcaccgctctcaacggcgtttcggcgtatgcatAGGAACTTATGAGCTCAGCC | Gibson primer for Sid regulator KO insert |
| AJS52 | CCTGGTCAGCTCGTCGAGCA | Diagnostic primer for deletion of Sid regulators;  Diagnostic primer for deletion of *sidA* |
| AJS53 | TCCTGCCTGTCTCGGTCACC | Diagnostic primer for deletion of Sid regulators |
| AJS56 | tgcatacgccgaaacgccgttgagagcggtgctcagccgaggcgtacggacctaggTGCAGCTCACGGTAACTGAT | Gibson primer for *sidA* KO insert |
| AJS57 | ttcggctgacaccacaggtactgggaggcgaaatgtgcggaatcaccggtcctaggAGGAACTTATGAGCTCAGCC | Gibson primer for *sidA* KO insert |
| AJS66 | CTTCGAGCTGAAGCGGACGC | Diagnostic primer for deletion of *sidA* |
| AJS67 | atcttgacggctggcgaga | Diagnostic primer for pIJ10257 MCS |
| AJS68 | ggcattgagcgtcagcata | Diagnostic primer for pIJ10257 MCS |
| AJS75 | tcggtcatcgctgtcctcgtttctggtggtactcaggcgaaggaagccggcctaggTGCAGCTCACGGTAACTGA | Gibson primer for *sidC* KO insert |
| AJS76 | cgggaagaccccagtgaagatcggagacgggaatgtccaacgacctcatgcctaggAGGAACTTATGAGCTCAGCC | Gibson primer for *sidC* KO insert |
| AJS77 | TCAGCCAACGCGAGGTCTGC | Diagnostic primer for deletion of *sidC* |
| AJS78 | CAGCCTCGTGCTCACCACCA | Diagnostic primer for deletion of *sidC* |
| AJS59 | tctagaacaggaggccccatATGGAATCCGCATCGATCAGGGAA | Gibson primer for *sidR1* complement insert;  Gibson primer for Sid-Regs complement insert |
| AJS60 | gagaacctaggatccaagctTCAAGCGCCCGGAAGCTCCA | Gibson primer for *sidR1* complement insert |
| AJS61 | tctagaacaggaggccccatATGACCGCCCGTGGCCTGAT | Gibson primer for *sidR2* complement insert |
| AJS62 | gagaacctaggatccaagctTCAGCGACCGGCCACGCA | Gibson primer for *sidR2* complement insert |
| AJS63 | tctagaacaggaggccccatATGCCACTTCGAGCGGCCAG | Gibson primer for *sidR3* complement insert |
| AJS64 | gagaacctaggatccaagctTCAACGGCGTTTCGGCGTGC | Gibson primer for *sidR3* complement insert;  Gibson primer for Sid-Regs complement insert |


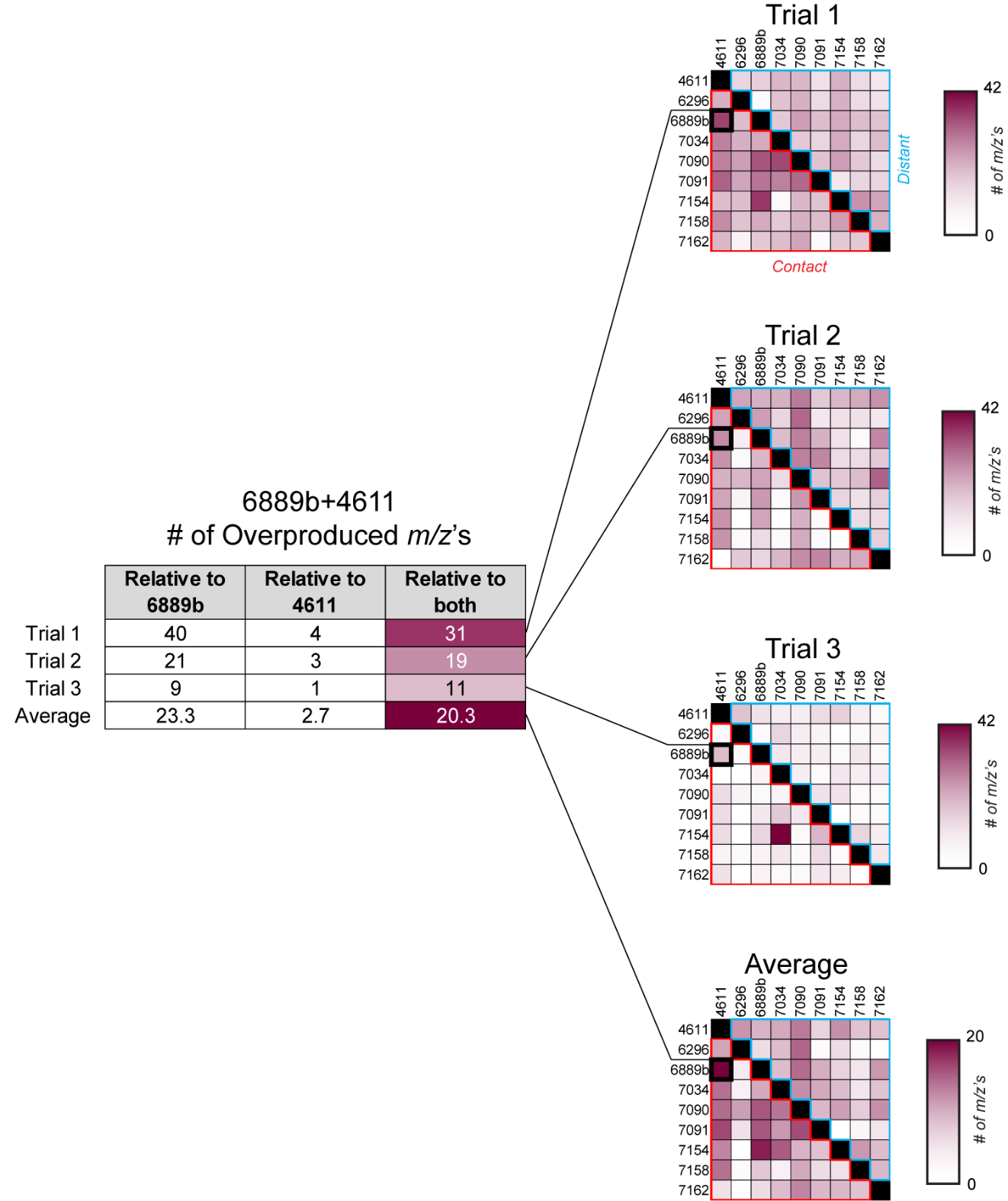


**Supplementary Figure S1: Heterogeneity in Coculture Replicates.** (*Left*) Number of m/z’s overproduced ≥ 10-fold in the WAC06889b+WAC04611 coculture condition relative to their constituent strains. (*Right*) Number of overproduced m/z’s for each coculture condition relative to both constituent strains. Each trial and the average of the three trials is shown. “Distant”: strains grown in the same well but at opposite ends of the well. “Contact”: strains mixed and grown in contact with each other.


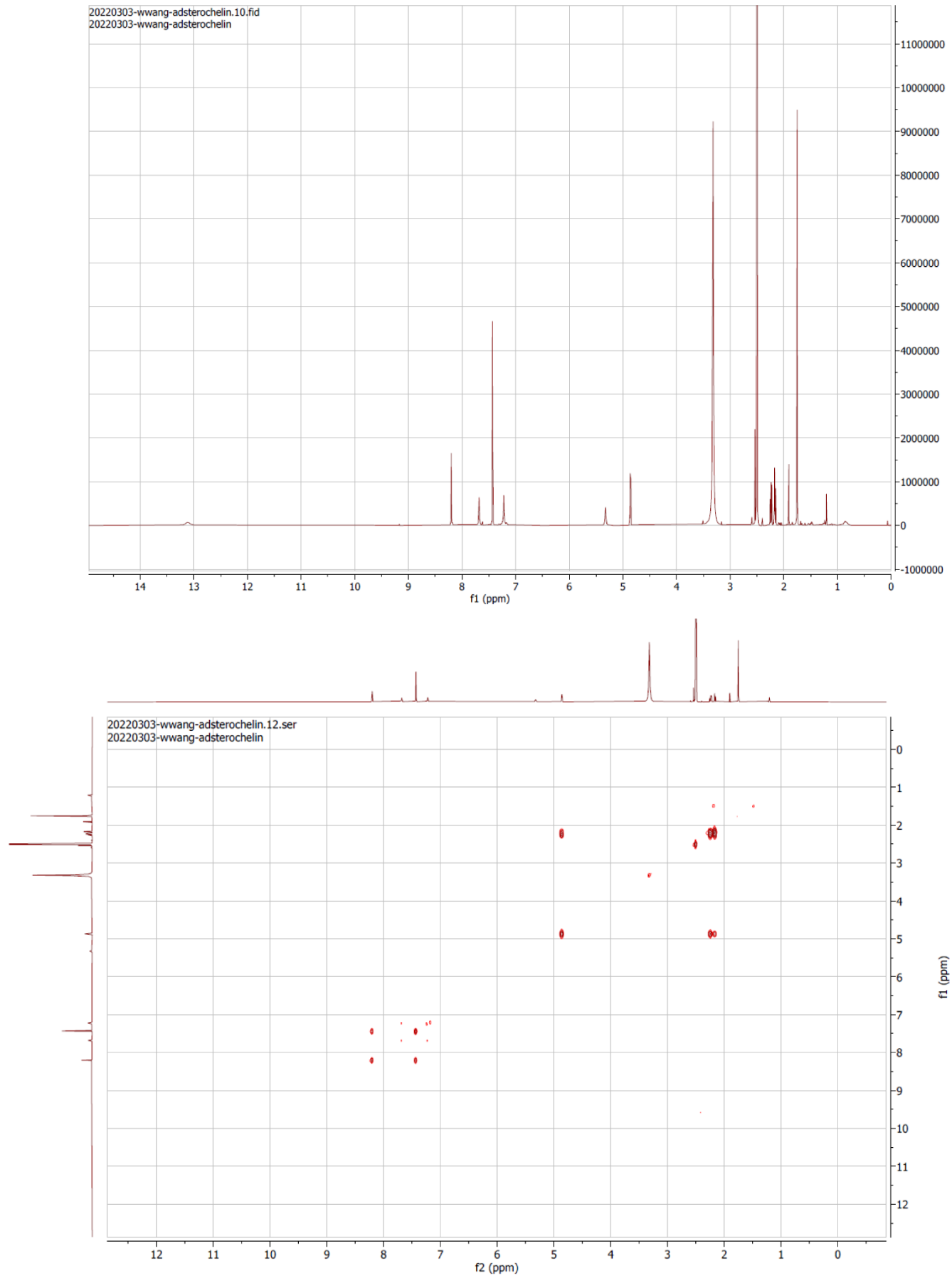


**Supplementary Figure S2: NMR Spectra for Siderochelin.** (*Top*) ^1^H NMR. (*Bottom*) COSY NMR


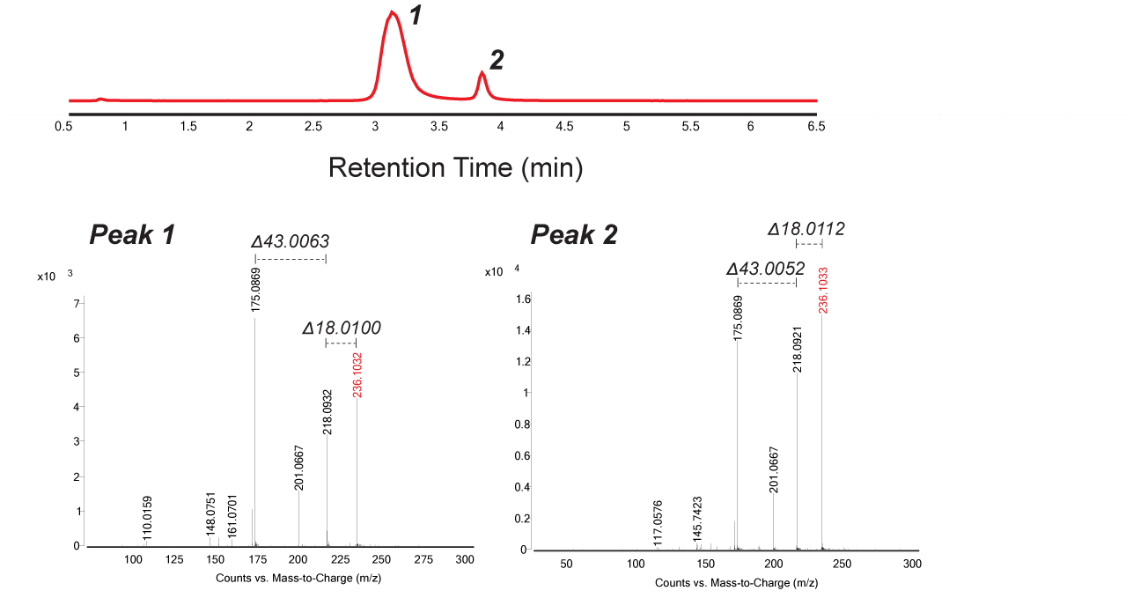


**Supplementary Figure S3: MS/MS Fragmentation of Siderochelin Peaks.** (*Top*) EIC trace of the m/z for siderochelin A/B. (*Bottom*) MS/MS fragmentation of peaks as denoted in the EIC trace. Both peaks have an identical fragmentation pattern.


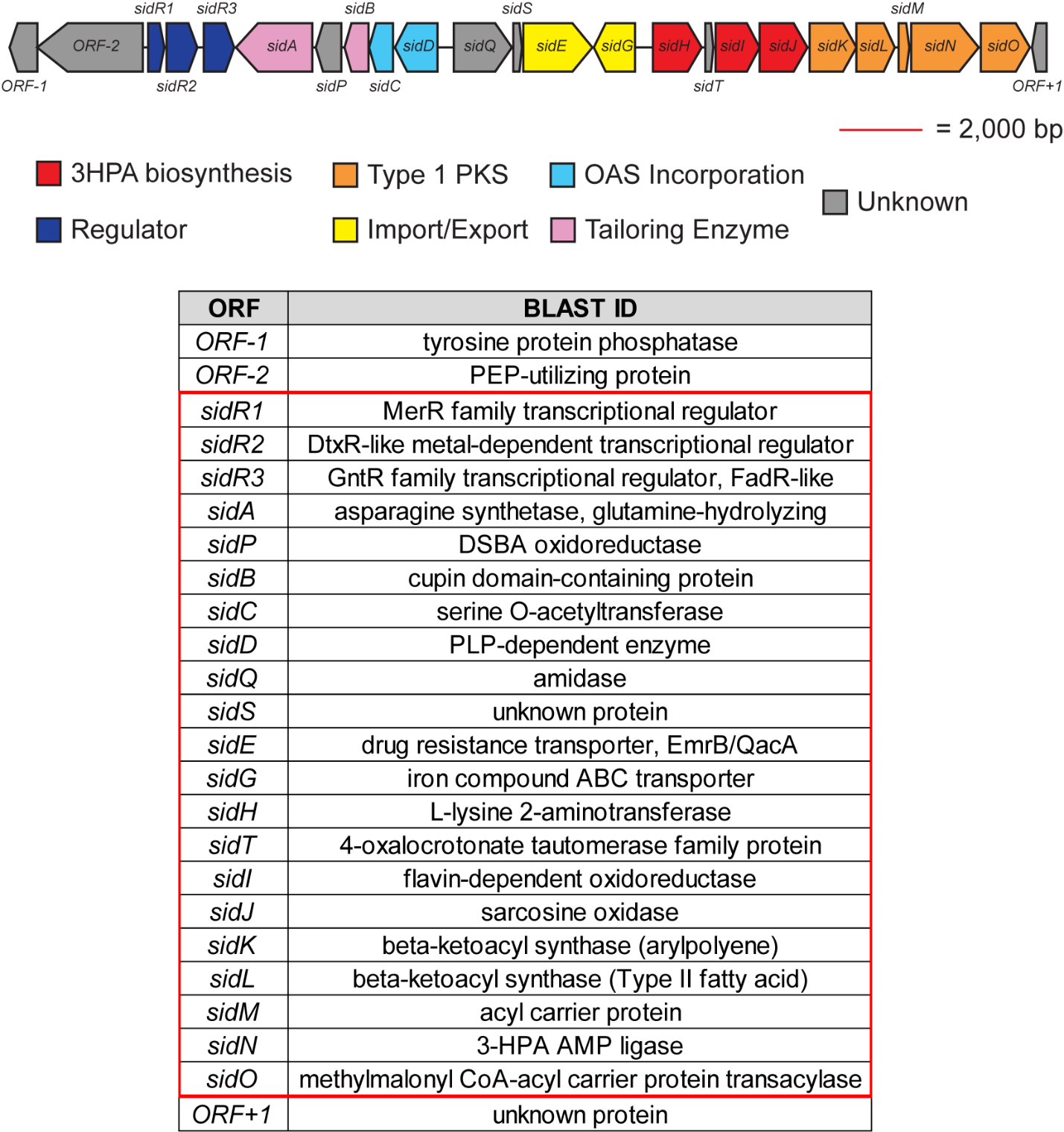


**Supplementary Figure S4: Captured Siderochelin BGC.** ORFs are colored based on predicted functions. The predicted BGC boundaries are denoted in the table by a red box.


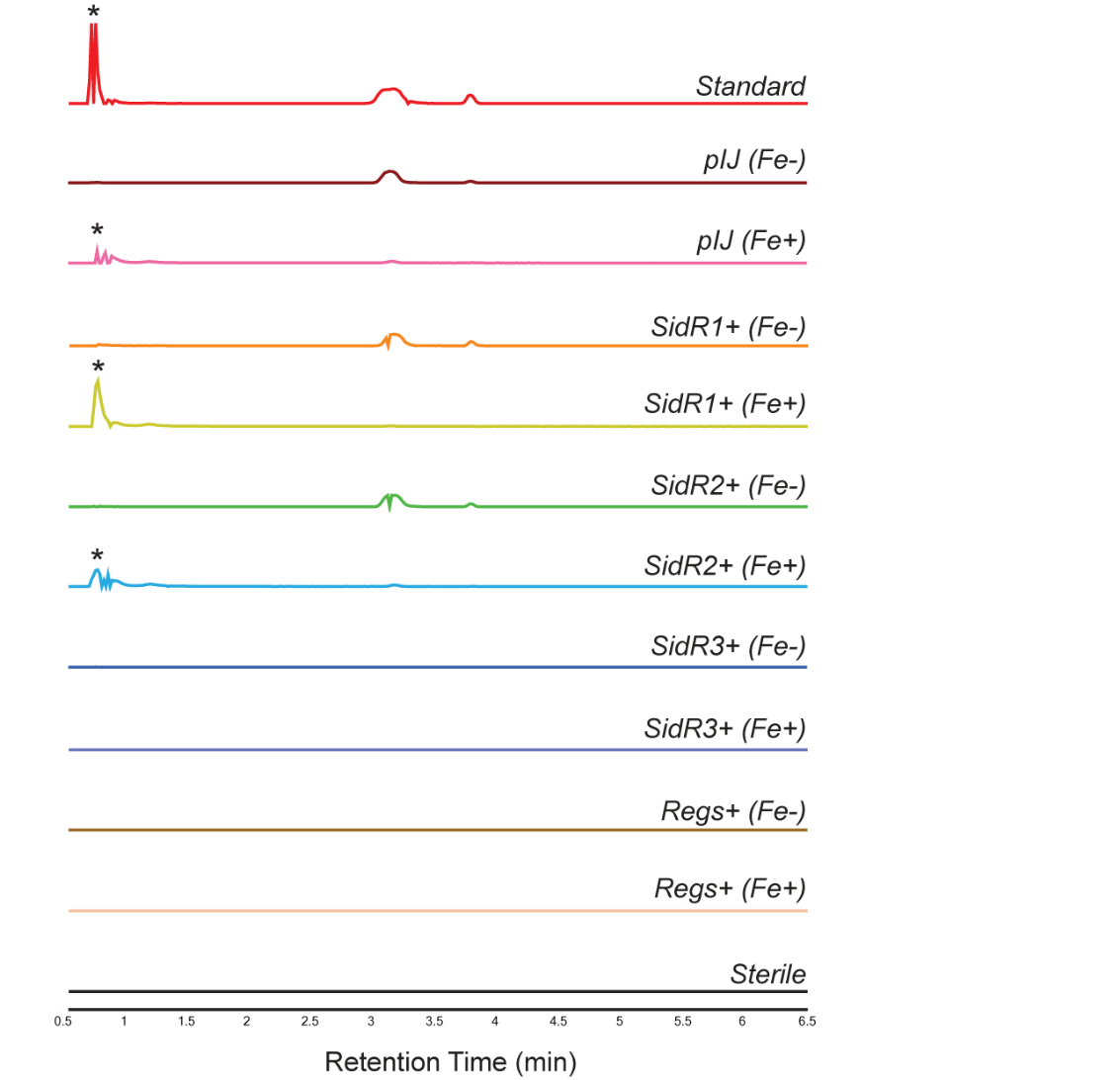


**Supplementary Figure S5: EIC Trace of Siderochelin-Iron Complex.** EIC for the m/z 525.1184 [(M-H^+^) x 2 + Fe^2+^ + H^+^]. Asterisk marks the presence of the siderochelin-iron complex. Traces are representative of 3 biological replicates.


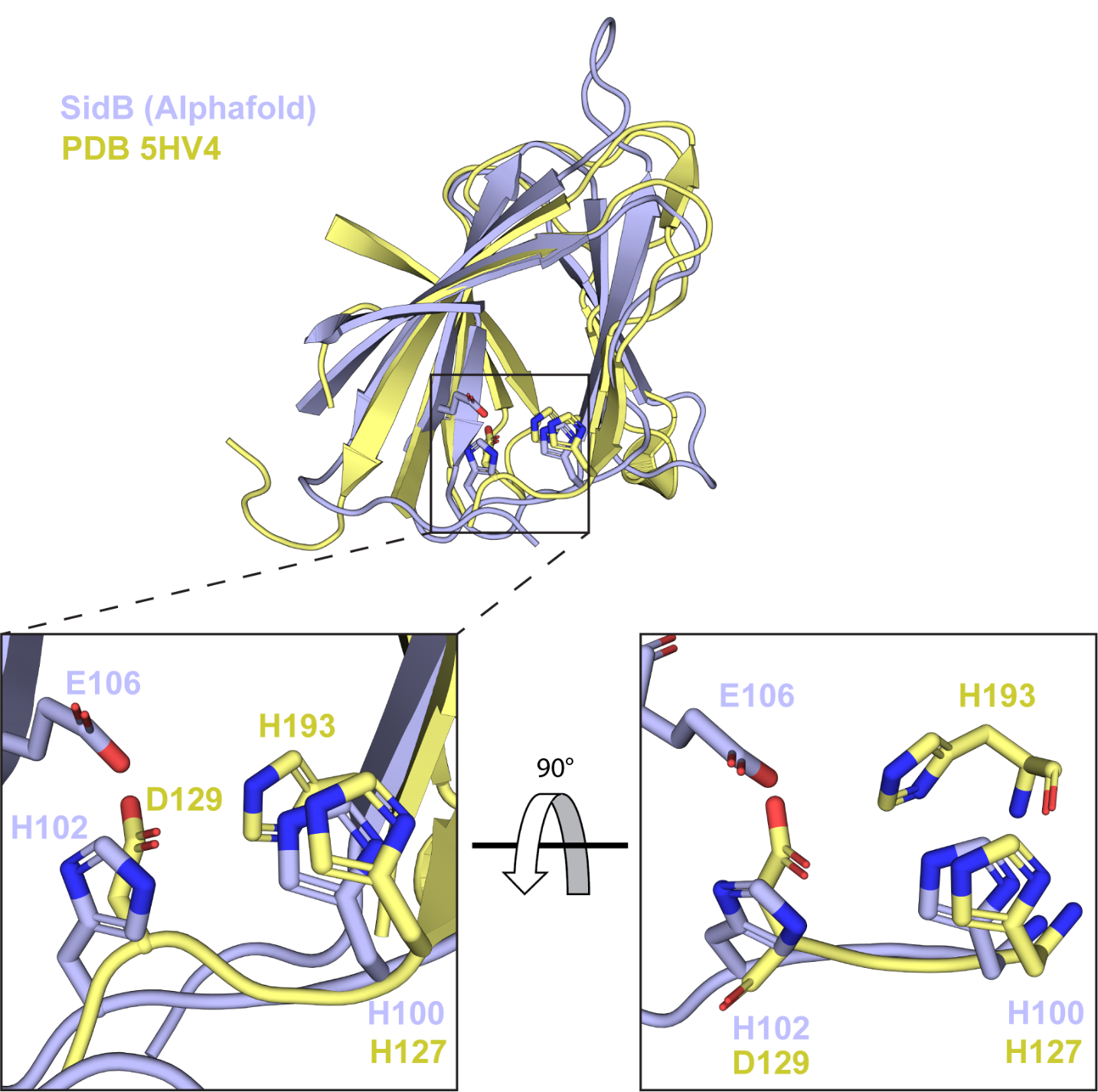


**Supplementary Figure S6: SidB Predicted to Possess a Noncanonical HxH…E Motif.** (*Top*) Alphafold model of SidB (lightblue) aligned to proline hydroxylase from *B. anthracis* (PDB 5HV4, paleyellow). (*Bottom*) Magnified view of the HxD…H motif of 5HV4. SidB motif appears rotated 90° to the right in 3D space relative to 5HV4. Loops are removed in all models for clarity.


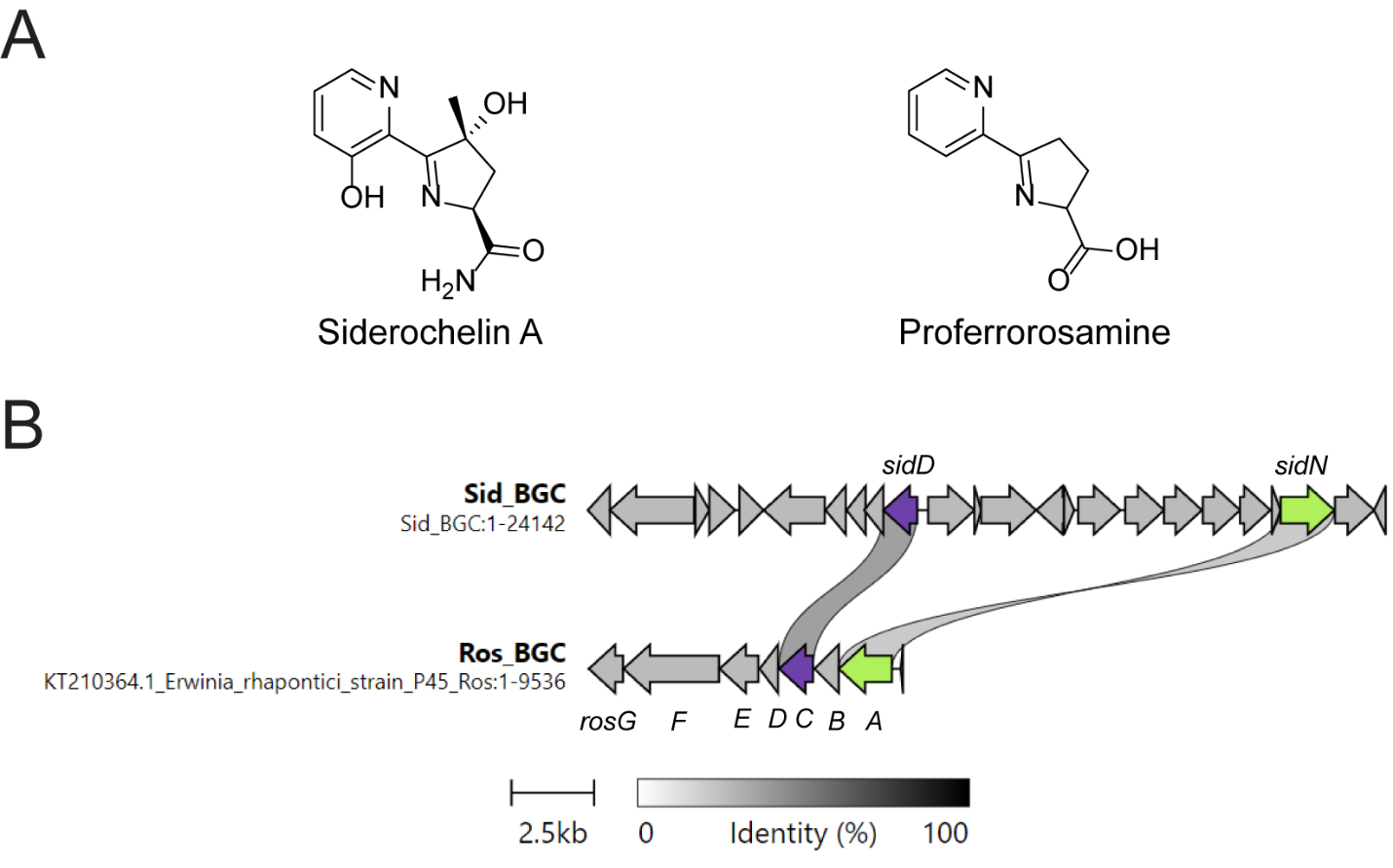


**Supplementary Figure S7: Siderochelin and Proferrorosamine BGCs Display Little Sequence Identity. A)** Structures of siderochelin A and proferrorosamine. **B)** comparison of the nucleotide sequences of the siderochelin BGC (“Sid_BGC”) and the proferrorosamine BGC (“Ros_BGC”). Only *sidD* and *sidN* show any sequence identity to their counterparts in the *ros* BGC.


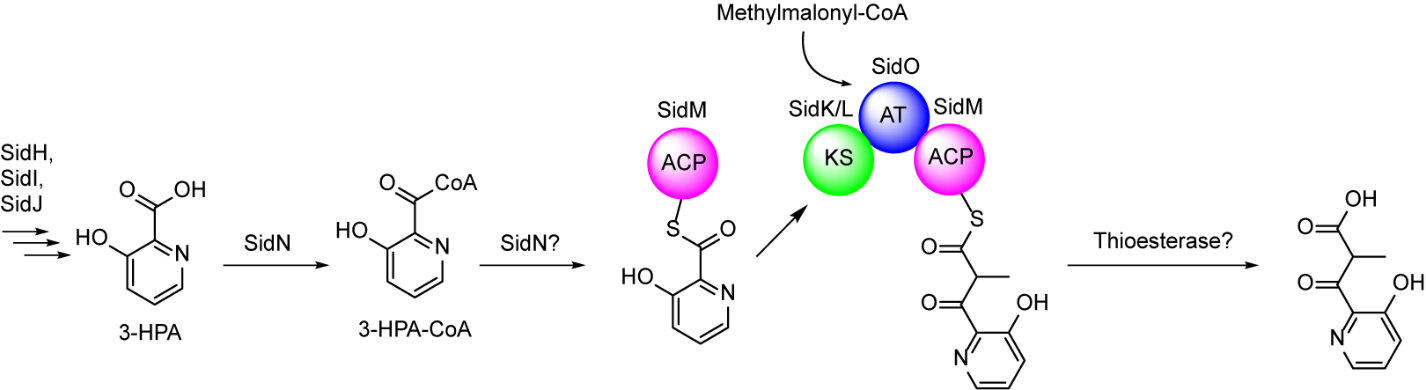


**Supplemental Figure S8: Proposed Model for the PKS steps in Siderochelin Biosynthesis.** 3-HPA is converted to 3-HPA-CoA by SidN then subsequently loaded onto SidM. The loaded 3-HPA is then transferred to the SidK/L-SidO-SidM complex where it is elongated by a single methylmalonyl subunit. An unknown thioesterase releases the product from the complex. ACP: acyl carrier protein, KS: ketosynthase, AT: acyltransferase


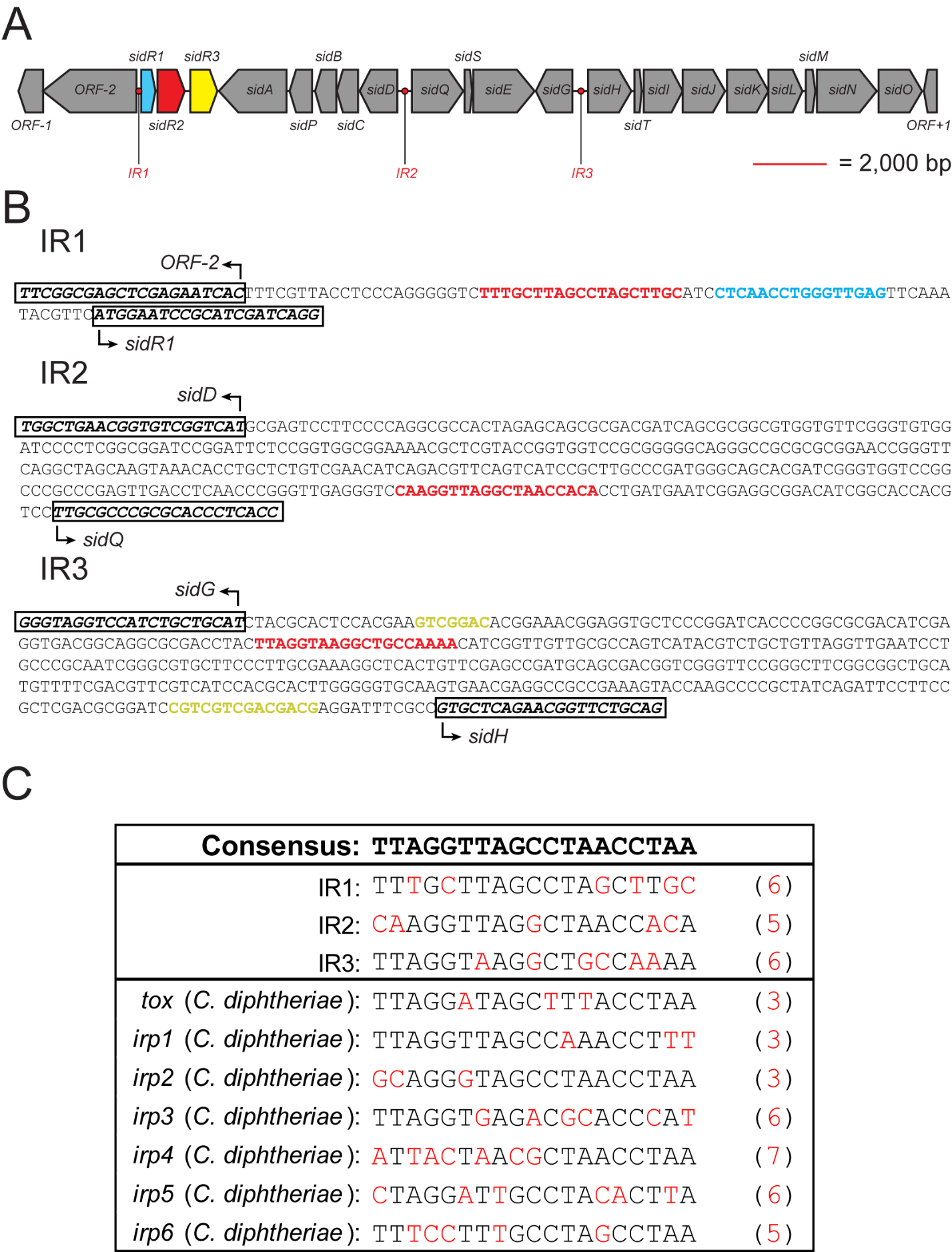


**Supplementary Figure S9: Potential binding sites for *sid* Regulators. A)** Schematic of the siderochelin BGC. Intergenic regions (IR) of interest are labeled 1-3. **B)** Sequences of intergenic regions of interest. ORF coding sequences are highlighted in black boxes. Potential DtxR-like binding sites are colored red, MerR-like binding sites in light blue, and FadR-like binding sites in yellow. **C)** Comparison of the three DtxR-like binding sites with the DtxR consensus sequence and known DtxR binding sites from *Corynebacterium diphtheriae*. Nucleotides which deviate from the consensus sequence are colored red and enumerated in parentheses.

***References***

1. Cox G, Sieron A, King AM, De Pascale G, Pawlowski AC, Koteva K, Wright GD. 2017. A Common Platform for Antibiotic Dereplication and Adjuvant Discovery. Cell Chem Biol 24:98–109.

2. Datsenko KA, Wanner BL. 2000. One-step inactivation of chromosomal genes in *Escherichia coli* K-12 using PCR products. Proc Natl Acad Sci 97:6640–6645.

3. Noskov V, Kouprina N, Leem S-H, Koriabine M, Barrett JC, Larionov V. 2002. A genetic system for direct selection of gene-positive clones during recombinational cloning in yeast. Nucleic Acids Res 30:8e – 8.

4. Gomez‐Escribano JP, Bibb MJ. 2011. Engineering *Streptomyces coelicolor* for heterologous expression of secondary metabolite gene clusters. Microb Biotechnol 4:207–215.

5. Tang X, Li J, Millán-Aguiñaga N, Zhang JJ, O’Neill EC, Ugalde JA, Jensen PR, Mantovani SM, Moore BS. 2015. Identification of Thiotetronic Acid Antibiotic Biosynthetic Pathways by Target-directed Genome Mining. ACS Chem Biol 10:2841–2849.

6. Hong H-J, Hutchings MI, Hill LM, Buttner MJ. 2005. The Role of the Novel Fem Protein VanK in Vancomycin Resistance in Streptomyces coelicolor. J Biol Chem 280:13055–13061.
